# Identification of novel variants underlying non-syndromic primary ovarian insufficiency using a targeted NGS gene panel

**DOI:** 10.3389/fendo.2025.1659701

**Published:** 2025-12-11

**Authors:** Claudia Veneziano, Jessica Parrotta, Daniela Lico, Gianluca Santamaria, Gemma Antonucci, Maria Teresa De Angelis, Fulvio Zullo, Giuseppe Viglietto, Carmela De Marco, Roberta Venturella

**Affiliations:** 1 Molecular Oncology Laboratory, Department of Experimental and Clinical Medicine, “Magna Graecia” University, Catanzaro, Italy; 2 Interdepartmental Center of Services (CIS), Omics and Biobank, “Magna Græcia” University, Catanzaro, Italy; 3 Unit of Obstetrics and Gynecology, Department of Experimental and Clinical Medicine, “Magna Graecia” University, Catanzaro, Italy

**Keywords:** non-syndromic primary ovarian Insufficiency (nsPOI), next generation sequencing (NGS), NGS panel, female infertility, genetic screening

## Abstract

**Background and objectives:**

Primary ovarian insufficiency (POI) affects 1-4% of women and is associated with infertility and reduced life expectancy. Most cases are idiopathic, and a genetic alteration is often the most plausible cause. In this study, we investigated whether targeted next-generation sequencing (NGS) analysis in combination with the OvAge^©^ method, which integrates biochemical and ecographic parameters, can reliably identify specific genetic variants underlying the occurrence of non-syndromic primary ovarian insufficiency (nsPOI).

**Methods:**

We enrolled 100 women with nsPOI and 200 healthy controls. A targeted NGS panel covering 72 genes potentially involved in POI was developed using Ampliseq technology (ThermoFisher Scientific). Various bioinformatic tools (Polyphen, Sift, CADD, MutationTaster and the Grantham score) were used to identify potentially pathogenic variants according to ACMG guidelines, while tools such as STRVCTVRE, CADD-SV and X-CNV were used to predict pathogenicity of CNV calls.

**Results:**

Using this panel, we identified mutations in 60% (N=60) of the patients, of whom 23% carried likely pathogenic or pathogenic mutations, and 37% had variants of uncertain significance (VUS). Among these 60 patients, 37 had monogenetic variants and 23 had mutations in two or more genes. In total, we identified 42 genes affected in our Italian of nsPOI cohort. The most frequently mutated genes in our cohort included DNAH5, LAMC1, ADAMTS1/19, HSD17B4, HK3 and AR. Additionally, we detected CNVs in the SYCE, DUSP22 and INHBB genes. Most of the altered genes in our cohort are involved in DNA repair, meiosis and signal transduction. Gene Ontology (GO) analysis revealed that the mutated genes play a key role in oocyte differentiation, folliculogenesis and follicular maturation.

**Discussion:**

Our main conclusion is that the development of a test integrating clinical, ultrasound, biochemical (OvAge©) and genetic data could substantially enhance early identification of women at risk of POI and offer opportunities for fertility preservation, such as oocyte cryopreservation or prioritizing reproductive efforts.

## Graphical Abstract

**Figure:**
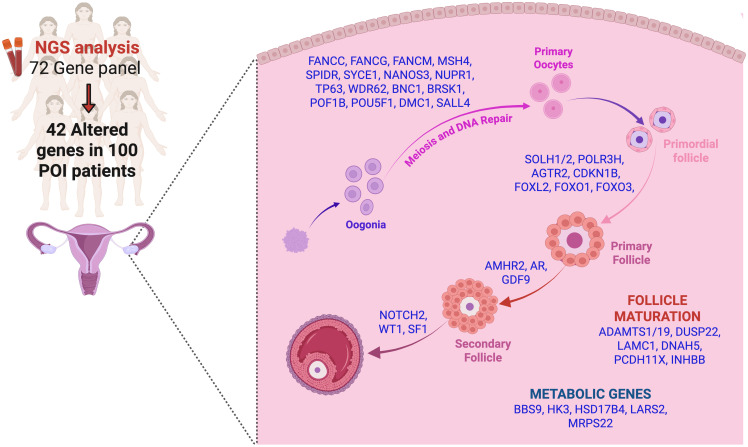

## Introduction

Primary ovarian insufficiency (POI) is a complex disease affecting approximately 1-4% of women worldwide (1). POI is characterized by amenorrhea in women under of the age of 40, hypoestrogenism, and elevated levels of follicle-stimulating hormone (FSH), leading to progressive loss of ovarian function and infertility (2–4) The long-term consequences of POI include an increased risk of osteoporosis, cardiovascular disease, and mental health disorders (5, 6). As such, POI has a substantial impact on women’s reproductive and overall health. The etiology of POI is heterogeneous and may result from various causes, including iatrogenic and environmental exposures (7, 8) autoimmune and metabolic diseases (9, 10), viral infection (11), X-linked abnormalities (12, 13) and autosomal gene mutations (14, 15).

To date, the genetic mechanism underlying POI remain poorly understood, no standardized diagnostic methods currently exist to detect its genetic basis. POI may present as part of a pleiotropic genetic syndrome or as an isolated, non-syndromic form. The advent of the “omics” era has led to the discovery of several genes implicated in both syndromic and non-syndromic POI due to their key roles in reproductive function (16–28).

Given the high genetic heterogeneity and relatively low prevalence of POI, traditional approaches such as candidate gene studies and genome-wide association studies (GWAS) are limited in their ability to elucidate the underlying genetic causes. While some pathogenic variants have been identified in specific families or subsets of patients, their contribution to the POI phenotype often remains uncertain. This knowledge gap delays the development of accurate diagnostic tools and personalized treatment strategies. To address these limitations, next-generation sequencing (NGS) has emerged as a powerful technology for the comprehensive analysis of genes involved in ovarian function (21).

Recent studies (29–32) have demonstrated the utility of targeted NGS panels for identifying potentially pathogenic variants, allowing the simultaneous analysis of multiple genes and improving diagnostic efficiency in terms of cost and turnaround time. Targeted NGS represents a promising strategy to investigate selected genes associated with ovarian biology and enhance clinical management through more precise etiologic diagnoses (33).

In this study, we analyzed the genetic profile of women with non-syndromic POI using a targeted Ampliseq panel targeting the exons of 72 genes, selected based on a previous systematic review conducted by our group (34). The aim was to investigate the genetic basis of POI in a cohort of Italian patients selected according to OvAge^©^ algorithm (35). The OvAge algorithm integrates clinical, hormonal, and ultrasound parameters into a mathematical model that yields a single value, expressed as “ovarian age.” Its main advantage is the combination of complementary markers, which reduces the variability and limitations of individual tests and provides an intuitive output that can be directly compared with chronological age. Our ultimate goal was to develop a diagnostic test that integrates genetic information for clinical use, supporting the early identification of women at risk of POI and enabling timely fertility preservation strategies.

## Materials and methods

### Patient cohort

A cohort of 100 women with non-syndromic POI was recruited from the OvAge database, maintained at the Department of Experimental and Clinical Medicine of the Magna Graecia University of Catanzaro. This database contains data on more than 1,000 women, including serum levels of anti-Müllerian hormone (AMH), follicle-stimulating hormone (FSH), estradiol (E2), three-dimensional antral follicle count (AFC), vascular index (VI), flow index (FI), and vascular flow index (VFI). For all patients, ovarian reserve had previously been estimated using our proprietary OvAge algorithm (35). This algorithm is a mathematical formula that combines biochemical and ultrasonographic parameters to produce a single interpretable value, referred to as “OvAge”—an estimate of ovarian age—calculated using the following linear equation: OvAge = 48.05 – 3.14 × AMH + 0.07 × FSH – 0.77 × AFC – 0.11 × FI + 0.25 × VI + 0.1 × AMH × AFC + 0.02 × FSH × AFC. The formula was generated through the use of a Generalized Linear Model (GzLM).

For this study, patients were selected from the database based on the following criteria: onset of oligomenorrhea or menopause before the age of 40, elevated FSH level (> 40 mU/L), estradiol (E2) < 20 pg/ml, anti-mullerian hormone (AMH) < 1 ng/ml. Additionaly, patients were included if the difference between their OvAge and chronological age exceeded 10 years. A control group of 200 healthy women with an OvAge–chronological age difference of ±2 years was also included. The following exclusion criteria were applied to both POI patients and controls: use of estrogens or progestins or breastfeeding within two months prior to enrollment, ongoing pregnancy, history of endometriosis, presence of ovarian follicles larger than 10 mm or other ovarian cystic lesions, chromosomal abnormalities, history of ovarian surgery, chemotherapy/radiotherapy, polycystic ovary syndrome, known autoimmune diseases, chronic, systemic, metabolic and endocrine pathologies, history of drug use. The sample size was estimated *a priori* using a two-sample test for proportions, according G*Power software to obtain >95% CI. All participants provided written informed consent.

### DNA extraction and quality assessment

Genomic DNA was extracted from peripheral blood samples using the PureLink^®^ Genomic Kit (Invitrogen, Carlsbad, CA, USA) following the manufacturer’s protocol. DNA quality and quantity were assessed using the Qubit Fluorometer (Invitrogen) and the 4200 Tape Station Instrument (Agilent Technologies, Inc, Santa Clara, CA, USA). Only high-quality DNA samples were used for subsequent library preparation and sequencing.

### Next generation sequencing

NGS was performed using the Ion AmpliSeq™ POI Panel on the Ion Torrent platform (Thermo Fisher Scientific, MA, USA). This custom targeted NGS panel provides complete exon coverage of 72 genes known to be associated with POI (34) (see details in **Supplementary File S1**). Library preparation was performed using10ng of genomic DNA measured with the Qubit 2.0 fluorometer (Thermo Fisher Scientific). Libraries were prepared both manually according to the Ion AmpliSeq Library Kit Plus and automatically on the Ion Chef™ instrument using the Ion AmpliSeq Kit for Chef DL8 (Thermofisher Scientific). Libraries were deemed suitable for sequencing after quality and quantity assessment. The concentration of each cDNA library, which was prepared manually, was determined on the Agilent 4200 system using the Agilent High Sensitivity DNA Assay (Agilent Technologies) according to the manufacturer’s recommendations. The concentration of the automatically prepared cDNA library pool was determined using the Ion Library TaqMan Quantitation Kit (Thermofisher Scientific). Libraries were diluted to 30 pM and then loaded into the Ion Chef™ instrument (Thermofisher Scientific). The Ion Chef™ instrument utilized the Ion 510™ & Ion 520™ & Ion 530™ Kit – Chef (Thermofisher Scientific) to perform emulsion PCR, enrichment and loading of the Ion S5–520 and/or 530 chip.

### Sanger sequencing

PCR products obtained with the BigDye Direct Cycle Sequencing Kit (Applied Biosystems, Thermofisher Scientific) were purified with the BigDye Terminator Purification Kit (Applied Biosystems) and sequenced with the BigDye Terminator v3.1 (Applied Biosystems, Foster City, CA, USA) using the Genetic Analyzer 3500 Dx (Applied Biosystems). The exons of DNAH5 (ex 66), LAMC1 (ex 17), AR (ex 1), GDF9 (ex 3), ADAMTS19 (ex 2) were amplified using the primer list in **Supplementary File S1**. The electropherograms were analyzed with SeqScape software, v.4 (Applied Biosystems) and compared with the corresponding reference genomes (DNAH5: NM_001369.3; LAMC1: NM_002293.4; AR: NM_000044.6; GDF9: NM_005260.7; ADAMTS19: NM_133638.6).

### Bioinformatic analysis

The raw data generated from NGS were processed using Torrent Suite software v.5.14 which performed sequence alignment, adapter trimming, signal filtering, and quality-based read exclusion. Coverage analysis was performed using the Coverage Analysis plug-in. The coverage criteria for removing sequences from further analysis were: Reads < 200,000; mean depth < 200; uniformity < 90% (see **Supplementary File S2** for details). Sequence variants detected within the 1549 amplicons of the 72 genes of the custom panel were analyzed with Ion Reporter version 5.18.2 using ‘Torrent Variant Caller v5.18-2 (Thermo Fisher Scientific). The parameters used to filter the variants were coverage, quality and frequency. The following parameters were set for the POI samples: Coverage ≥ 200, Quality score ≥ 30.

To remove common germline variants, variants detected in the POI cohort were filtered against those found in the control group (N = 200). The POI candidate variants were further filtered by the non-Finnish European population from GnomAD (https://gnomad.broadinstitute.org/) and the European population from 1000 Genomes (https://www.internationalgenome.org/). The potential deleterious effects of the identified variants were assessed using the prediction algorithms SIFT and Polyphen2 (36), CADD (37), Grantham (38), MutatioTaster (39). Finally, the semi-automated process of the InterVar web service (40) and a manual review were used to identify the resulting potentially pathogenic variants. Both methods adhered to the American College of Medical Genetics and Genomics (ACMG) guidelines (41). The criteria used based on Richards et al. are listed in **Supplementary File S3**.

### Copy number variation analysis

CNV analysis was performed using Ion Reporter software version 5.18.2. Briefly, a Hidden Markov Model (HMM)-based algorithm used normalized read coverage across amplicons to predict ploidy values (0, 1, 2, 3, etc.). Prior to CN determination, read coverage is corrected for GC bias and compared to a baseline coverage created from a control group of regions with known ploidy status. For each sample, a MAPD (Median Absolute Pairwise Difference) value is calculated, a metric that measures the noise in read coverage across all amplicons. To create a CNV call, MAPD is <0.4 was used as criteria. We considered the confidence score that filters out CNV regions that are likely to be false positives (42). As recommended by Ion Reporter software, we considered significant only CNVs that showed confidence score > 10. According to commonly used parameters (43), focal CNVs were defined as aberrations <3Mb in size.

Three supervised learning-based tools (STRVCTVRE (https://strvctvre.berkeley.edu/), CADD-SV (https://cadd-sv.bihealth.org/) and X-CNV (http://119.3.41.228/XCNV/search.php) were used to predict the impact of CNV on pathogenicity. These predictors for the effect of structural variation (SV), CADD-SV (44) and StrVCTVRE (45), are trained with a random forest classifier and use a set of genomic features for SVs related to conservation, gene importance, coding region, expression and exon structure. X-CNV (46) is a framework based on the probabilistic value of the XGBoost algorithm that generates a meta-voting prediction (MVP) score to quantitatively measure the pathogenic effect of CNVs. As stated by the developers (44), the CADD-SV score on the Phred scale ranges from 0 (potentially benign) to 48 (potentially pathogenic). The StrVCTVRE score ranges from 0-1, with a score of 1 being more harmful (45). The MVP score of the X-CNV tool (46) indicates that CNVs between 0.46 and 0.76, between 0.16 and 0.46 and between 0.14 and 0.16 have potentially likely pathogenic, uncertain and likely benign effects, respectively.

### Q-RT-PCR validation

Genomic DNA was prepared using standard methods. Quantitative real-time PCR (Q-PCR) was performed using the Power SYBR Green PCR Master Mix with the QuantStudio 12K Flex Real Time System (ThermoFisher), as previously described (47). Normalization was performed to the GAPDH DNA content. Relative DNA amounts were calculated using the comparative cycle threshold method (48). Primer sequences are listed in **Supplementary File S1**. Statistics was performed by Student’s t-test.

## Results

### Sequencing analysis of POI patients

**Figure 1:**
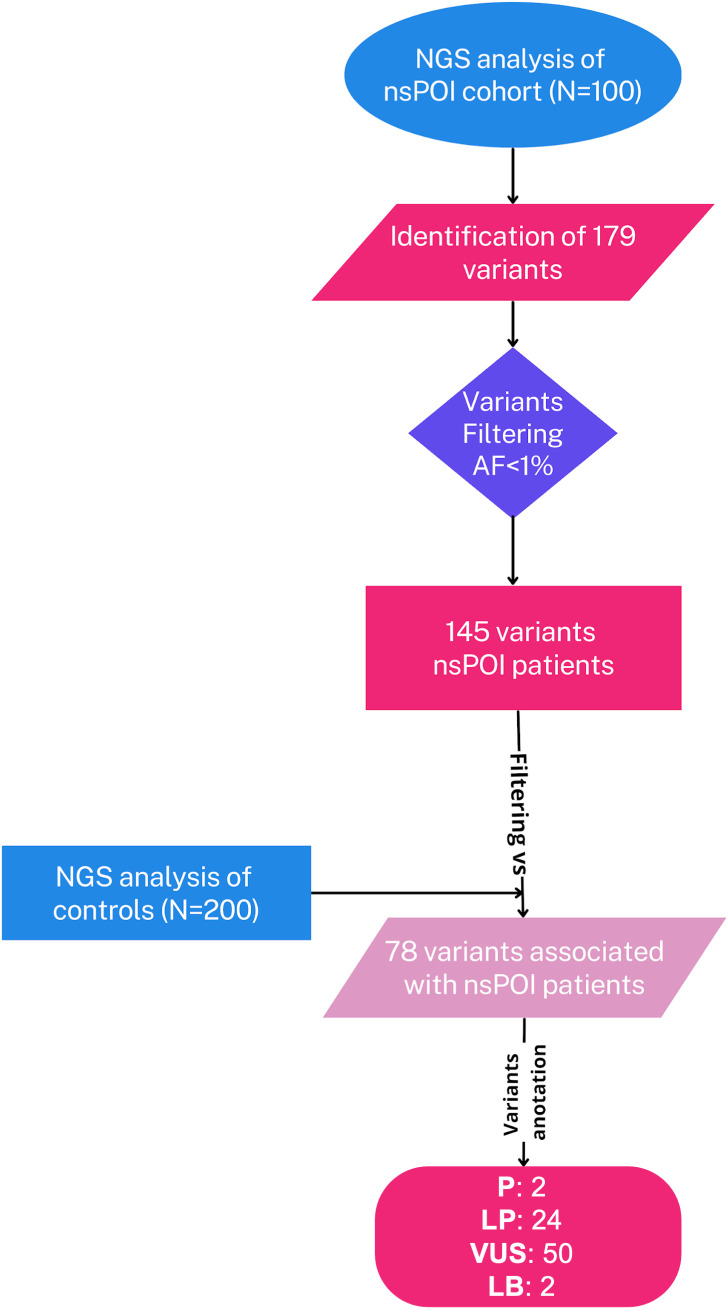
Workflow of the NGS analysis. The flowchart shows the workflow to identify the potential causative genetic variants in our nsPOI patients. Legend: AF, allele frequency; P, pathogenic; LP, likely pathogenic; VUS, variant of uncertain significance; LB, likely benign.

The aim of this study was to characterize the mutational profile of individuals affected by non-syndromic primary ovarian insufficiency (hereafter referred as nsPOI). To this end, we sequenced all coding exons of 72 genes using the Ampliseq technology on the Ion Torrent platform (**Supplementary File S1**). The experimental workflow is outlined in **Figure 1**.

Genomic DNA from peripheral blood samples of 100 Italian women with nsPOI and 200 healthy controls was sequenced (see Materials & Methods for inclusion and exclusion criteria). Variants were filtered to exclude those with a frequency greater than 1% or those not present in the non-Finnish European population of the gnomAD and 1000 Genomes databases (**Figure 1**).

**Figure 2:**
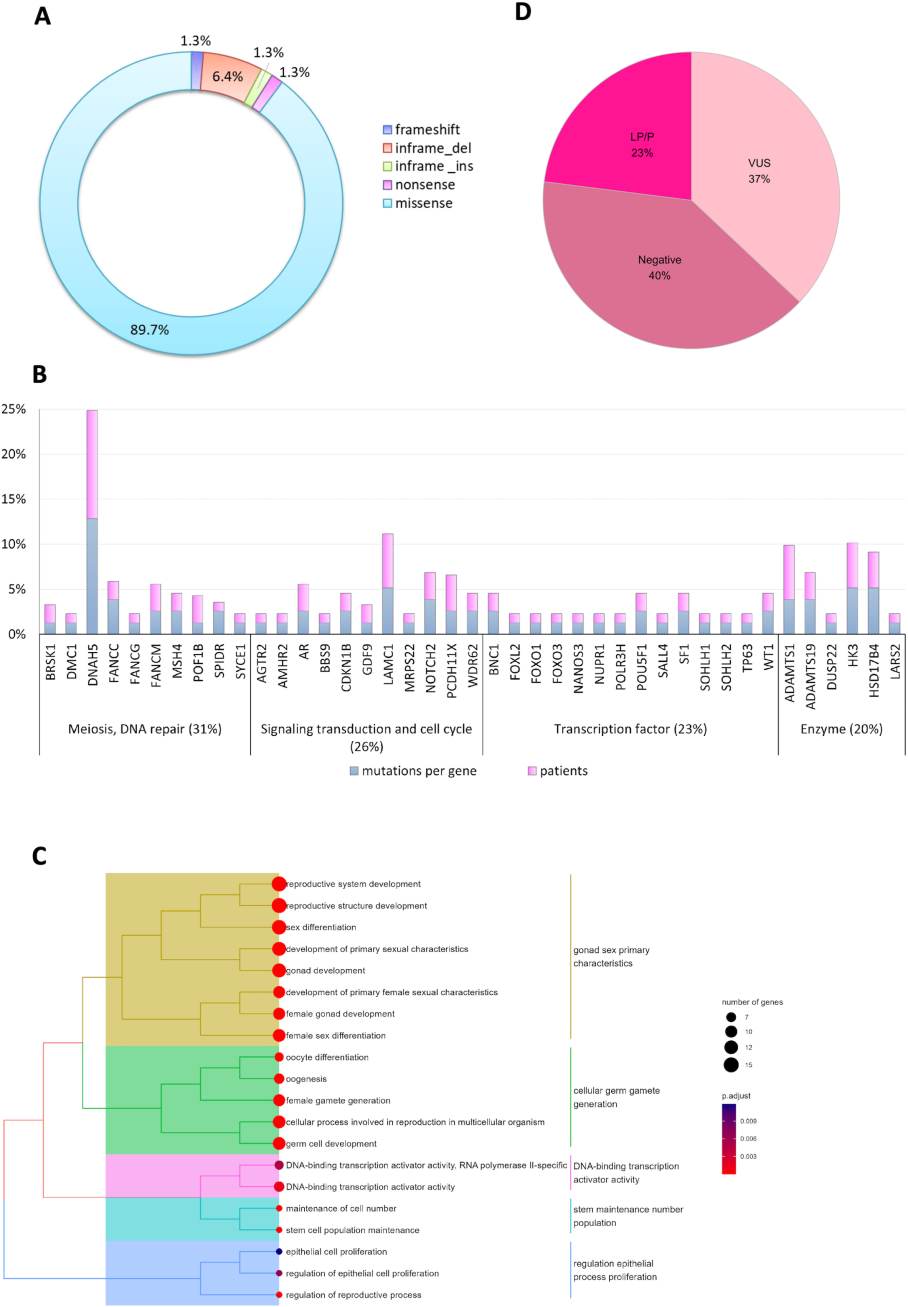
Genomic characteristics of the nsPOI cohort. **(A)** Pie chart showing the frequency of each mutation type identified in nsPOI patients. **(B)** Histogram displaying the prevalence of variants per gene. The 78 filtered variants were found in 41 genes, encoding (from left to right): 10 meiotic factors, 11 proteins related to signal transduction/cell cycle, 14 transcription factors, and 6 enzymes. The y-axis indicates the frequency of variants in each gene (blue) and the percentage of patients carrying variants in that gene (pink). Percentages of each gene class correspond to the sum of blue bars. **(C)** Tree plot showing hierarchical clustering of positively enriched GO terms among the 41 mutated genes. Circle size reflects the number of genes per GO term; circle color indicates adjusted p-value significance. Clusters are annotated with representative keywords. **(D)** Pie chart presenting the proportion of patients with LP/P or VUS variants versus those with no detectable mutations (negative).

By comparing the nsPOI cohort with healthy controls (**Supplementary File S4**), we identified 78 unique variants (**Supplementary File S5**) that were exclusively present in 60 of the 100 nsPOI patients. The remaining 40 patients did not carry any mutation in the analyzed genes (**Figure 1**). These variants included 70 missense (89,7%), 1 nonsense (1,3%), 1 frameshift (1,3%), 5 in-frame deletions (6,4%) and 1 in-frame insertion (1,3%) variants (**Figure 2A**). These 78 variants were distributed across in 41 genes (**Supplementary File S5**) encoding 14 transcription factors, 11 proteins involved in signal transduction and the cell cycle, 10 meiotic factors and 6 enzymes (**Figure 2B**). Genes involved in meiosis showed the highest mutation frequency (31%), followed by those involved in signal transduction and cell cycle (26%), transcriptional regulation (23%) and enzymatic activity (20%). The Gene Ontology analysis of the mutated genes is presented in **Figure 2C** and **Supplementary File S6**. The most enriched functional categories (those including >6 genes) were related to the development of the female reproductive system and gamete generation, although many genes were also involved in transcriptional regulation and maintenance of the stem cell niche.

To classify the identified variants, we followed the ACMG guidelines (41), using five predictive algorithms (SIFT, PolyPhen-2, MutationTaster, CADD, and Grantham) for optimal interpretive accuracy (see Materials and Methods and **Supplementary File S3** for details). Variants were classified as likely benign/benign (LB/B), likely pathogenic/pathogenic (LP/P) and of uncertain significance (VUS). In total, we identified 2 LB variants, 24 LP variants, 2 P variants, 50 VUS variants. Overall, 40% of patients were mutation-negative, while 60% carried at least one variant. Among these, 23% had LP/P variants and 37% had only VUS (**Figure 2D**). Representative Sanger sequencing validations of selected mutations are shown in **Supplementary Figure S1**.

### Genes mutated in a cohort from southern Italy

#### The most frequently mutated genes in our cohort

**Figure 3:**
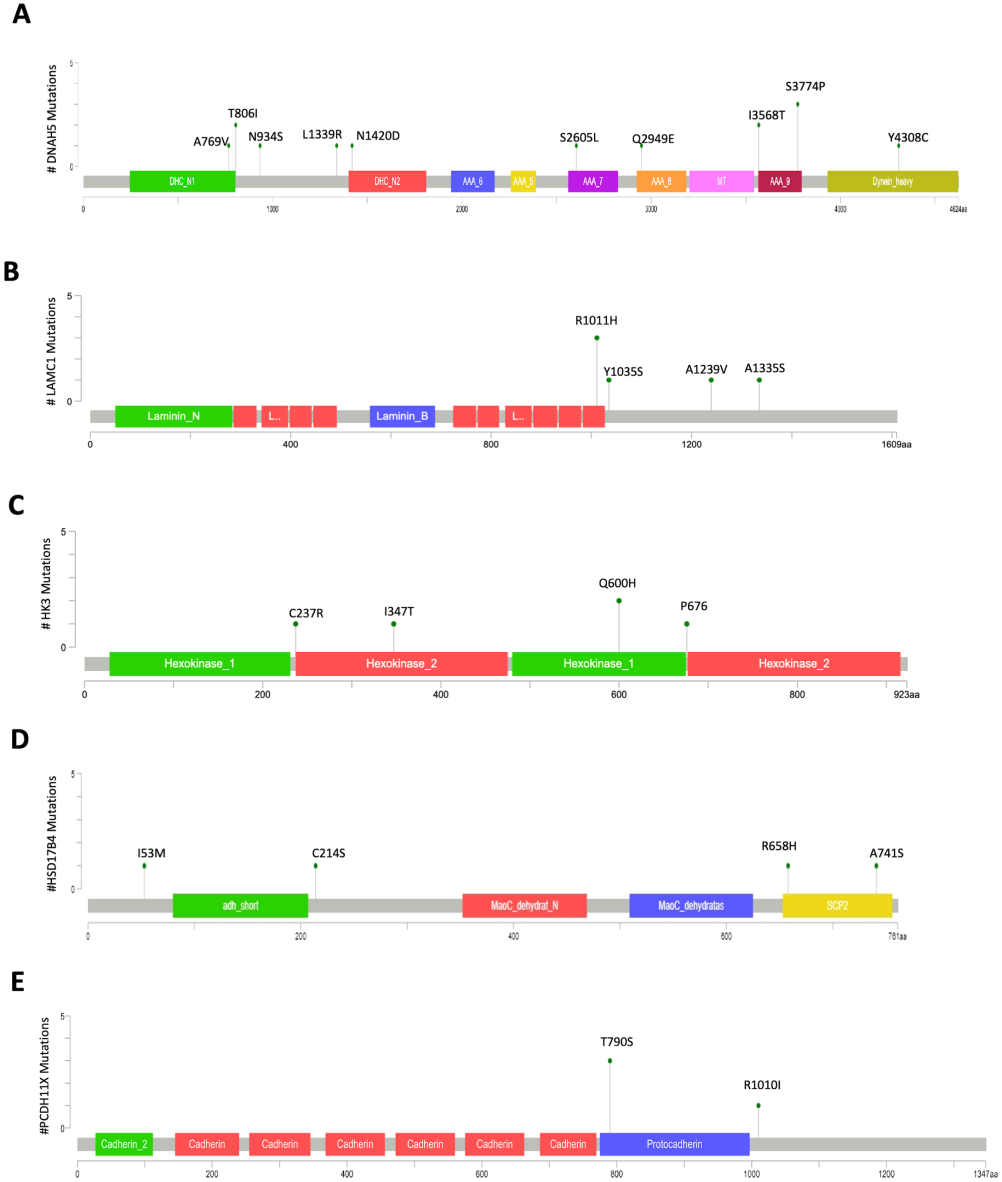
Mutation mapper plots of the most frequently mutated genes in nsPOI patients. The plots show the distribution of variants in *DNAH5***(A)**, *LAMC1***(B)**, *HK3***(C)**, *HSD17B4***(D)**, and *PCDH11X***(E)**. Amino acid changes are indicated above each lollipop, and line length represents the number of patients carrying the corresponding variant. Plots were generated using the Mutation Mapper tool on the cBioPortal platform (https://www.cbioportal.org/mutation_mapper).

The protein Dynein axonemal heavy chain 5 encoded by the *DNAH5* gene was mutated in twelve patients (12%). Three patients carried the S3774P variant, two had I3568T, two patients carried the variant T806I. Seven additional patients each presented a distinct mutation: A769V, S2605L, N1420D, L1339R, N934S, Q2949E, and Y4308C. Notably, S3774P and I3568T co-occurred in two patients. Except for T806I, N934S, L1339R, and I3568T, which were located in linker domains, the remaining mutations resided in conserved functional domains (**Figure 3A**). Mutations A769V and N1420D were located in the N-terminal region 1 (DHC_N1) and region 2 (DHC_N1) of the dynein heavy chain, respectively; mutations S2605L and Q2949E were located in the P-loop containing the dynein motor region D3 (AAA_7) and D4 (AAA_7), respectively; mutation S3774P was located in the ATP-binding dynein motor region D5 (AAA_9); and Y4308C was located in the dynein heavy chain region D6 of the dynein motor (**Figure 3A**). Mutations S2605L and N934S were classified as likely pathogenic, while the remaining mutations were categorized as variants of uncertain significance (VUS) (**Table 1**).

**Table 1:** Variants identified by targeted NGS in our POI cohort.

N. of patients	Gene	Type of mutation	AA change	gnomAD_NEF	EUR_AF	SIFT	PolyPhen	CADD	Mutation taster	Grantham	ACMG criteria	Classification
3	*DNAH5*	missense	S3774P	0.00013	NA	0	0.529	27.8	B	74	PP2, PP3, PM2, PM1	LP
2	*DNAH5*	missense	I3568T	0.00881	NA	0.23	0.001	21.9	B	89	PP2, PM2, PM1	VUS
1	*DNAH5*	missense	Q2949E	0.00315	0.002	0.08	0.002	21.4	D	29	PP2, PM2, PM1	VUS
1	*DNAH5*	missense	S2605L	NA	NA	0.08	0.028	23.7	D	145	PP2, PP3, PM6, PM1, PM2	LP
1	*DNAH5*	missense	N1420D	0.00041	0.0061	0.28	0.005	22.8	B	23	PP2, PM2, PM1, PM2	VUS
1	*DNAH5*	missense	L1339R	0.00019	0.002	0.6	0	18.98	B	102	PP2, PM2	VUS
1	*DNAH5*	missense	N934S	NA	NA	0.89	0	0.11	B	46	PP2, PM2	VUS
2	*DNAH5*	missense	T806I	0.01763	NA	0.28	0	15.24	B	58	PP2, PM2	VUS
1	*DNAH5*	missense	A769V	0.07048	NA	1	0	0.42	B	64	PP2, PM2	VUS
1	DNAH5	missense	Y4308C	0.09694	NA	0.05	0.03	18.84	B	194	PP2, BS1, BP4, BP6	LB
3	*LAMC1*	missense	R1011H	0.00078	0.001	0.07	1	27.1	D	29	PP2, PM5, PM2, PM1	LP
1	*LAMC1*	missense	Y1035S	NA	NA	0	1	28.4	D	144	PM2, PM5, PP2, PP3	LP
1	*LAMC1*	missense	A1239V	NA	NA	0.37	0.858	17.33	B	64	PM2, PP2	VUS
1	*LAMC1*	missense	A1335S	NA	NA	0.01	0.999	23.3	B	99	PP2, PP3, PM2, PM6	LP
1	*HSD17B4*	missense	I53M	NA	NA	NA	NA	5.95	B	10	PM6, PM1, PM2	LP
1	*HSD17B4*	missense	C214S	0.00004	NA	0.04	0.217	28.4	NA	112	PP3, PM2	VUS
1	*HSD17B4*	missense	R658H	NA	NA	0.03	0.984	23.6	B	103	PP3, PM1, PM2	VUS
1	*HSD17B4*	missense	A741S	NA	NA	0	0.752	26.5	B	99	PP3, PM6, PM1, PM2	LP
1	*HK3*	missense	P676S	0.00021	0.001	0.02	0.588	18.49	B	74	PP2, PP3, PM2	VUS
2	*HK3*	missense	Q600H	0.01082	0.008	0.15	0	19.02	B	24	PP2, PM2, PM1	VUS
1	*HK3*	missense	I347T	0.00011	NA	0	0.904	23.8	B	89	PP2, PP3, PM2, PM1	LP
1	*HK3*	missense	C237R	NA	NA	0	1	24.1	D	180	PM2, PM5, PP2, PP3	LP
3	*PCDH11X*	missense	T790S	0.03674	NA	0.08	0.035	5.332	B	32	PM2, PM1, PP2	VUS
1	*PCDH11X*	missense	R1010I	0.04897	NA	0	0.999	23.2	B	64	PM1, PM2, PP3, PP2	LP
2	*AR*	deletion	Q74_Q80del	NA	NA	NA	NA	13.48	D	NA	PM4, PM1, PM2	LP
1	*AR*	deletion	Q66_Q80del	NA	NA	NA	NA	13.48	D	NA	PM4, PM1, PM2	LP
1	*NOTCH2*	missense	I1693T	NA	NA	0.01	0.039	22.8	B	32	PM2, PP2	VUS
1	*NOTCH2*	missense	R318L	0.01764	NA	0.12	0.001	22.4	B	102	PM2, PM1, PP2	VUS
1	*NOTCH2*	missense	A147S	0.00885	NA	0.34	0.932	22.7	B	99	PM1, PM2, PP2, PP3	LP
1	*ADAMTS19*	missense	R64C	NA	NA	0	0.959	18.07	B	180	PM1, PM2, PP2, PP3	LP
1	*ADAMTS19*	missense	L117V	NA	NA	0.02	0	12.17	B	32	PM1, PM2	VUS
1	*ADAMTS19*	missense	G202S	0.00018	NA	0.35	0.005	13.87	B	56	PM1, PM2, BS1, BP4, BP6	LB
1	*FANCM*	missense	A48D	NA	NA	0.01	0.068	14.8	B	126	PM6, PM1, PM2	LP
1	*FANCM*	missense	L57F	0.00282	0.002	0.01	0.068	14.75	B	126	PM2, PM1	VUS
1	*FANCM*	missense	P1255L	NA	NA	0	0.078	na	B	22	PM6, PM1, PM2	LP
1	*FANCC*	missense	E273Q	0.01759	NA	0.01	0.993	25.3	B	29	PM2, PM1, PP3	VUS
1	*FANCC*	frameshift	I121Tfs*7	NA	NA	NA	NA	32	NA	NA	PVS1, PM2, PM1	P
2	*GDF9*	missense	R454C	0.0041	0.004	0	1	28.8	B	180	PS1, PM2, PM1, PP3	LP
1	*SPIDR*	nonsense	S64*	NA	NA	NA	NA	26.9	NA	NA	PVS1, PM2, PM1	P
1	*SPIDR*	missense	R294K	0.00051	0.0031	1	0.047	17.5	B	26	PM2, PM1	VUS
1	POU5F1	missense	T116S	NA	NA	0.11	0.062	6.86	B	58.0	PM2, PP2	VUS
1	POU5F1	missense	P65R	NA	NA	0.0	0.495	11.43	B	103.0	PM6, PM2, PP2	VUS
1	SF1	missense	G517A	0.01772	NA	0.02	0.998	24.8	B	60	PM2, PP2, PP3	VUS
1	SF1	deletion	G39_P47del	NA	NA	NA	NA	19.52	NA	NA	PM2, PM4, PM6, PP2	LP
1	*SOHLH1*	deletion	G323del	0.01191	NA	NA	NA	11.44	NA	NA	PM6, PM2, PM4	LP
1	*AMHR2*	missense	G153R	NA	NA	0	1	25.7	B	125	PM6, PM1, PM2, PP3	LP
1	*TP63*	missense	R487C	0.00019	NA	0	0.997	31	D	180	PM2, PM1, PM5, PP2	LP
1	*NANOS3*	duplication	G171dup	NA	NA	NA	NA	14.8	NA	NA	PM1, PM2, PM4	LP
1	*MRPS22*	missense	V23L	NA	NA	0.02	0.043	9.9	B	32	PM1, PM2, PM6	LP
1	*FOXL2*	deletion	A234del	0.00107	NA	NA	NA	16.23	NA	NA	PM1, PM2, PM4	LP

The table shows the most frequent variants identified in 60/100 patients in our cohort. For each variant the allele frequency in population databases (GnomAD, 1000 genomes_EUR), the pathogenicity prediction scores (Sift, Polyphen, Mutation Taster, CADD, Grantham) and the final classification according to ACMG criteria are reported. Abbreviations: GnomA NEF, Genome Aggregation Database Non-Finnish European; Eur AF, European population Allele Frequency; B, benign; D, deleterious; NA, not available; P, pathogenic; LP, likely pathogenic; VUS, variant of uncertain significance; LB, likely benign.

Six patients had 4 missense mutations (R1011H, Y1035S, A1239V, A1335S) in the protein encoded by the laminin subunit gamma 1 (*LAMC1*) gene. The R1011H mutation, which is localized in the laminin EGF domain (**Figure 3B**), was present in 3 patients. All mutations had a very low frequency or were not present in the public control databases, and their prediction scores supported potential pathogenicity at different levels (**Table 1**).

The gene encoding hexokinase 3 (*HK3*) was mutated in five patients carrying 4 missense mutations of the gene (C237R, I347T, Q600H, P676S). The mutations C237R and I347T are located in the hexokinase 2 domain, while the mutation Q600H is located in the hexokinase 1 domain and the mutation P676S is located in the junction domain between the hexokinase 1 and hexokinase 2 domains at the C-terminus (**Figure 3C**). In particular, C237R and I347T are likely pathogenic variants (**Table 1**).

Four patients had four different missense mutations of the *HSD17B4* gene encoding 17-beta-hydroxysteroid dehydrogenase 4, which is involved in the peroxisomal beta-oxidation pathway for fatty acids. The I53M and C214S mutations were located in non-functional domains, while the R658H and A741S missense mutations were located in the sterol carrier protein 2 (SCP2) domain, which is responsible for the binding and transfer of sterols and phospholipids (**Figure 3D**). The mutations I53M and A741S were classified as likely pathogenic variants (**Table 1**). In our cohort, four patients had missense mutations in PCDH11X. The mutation T790S, located in the protocadherin domain, was found in three patients and R1010I, at the C-terminus of the protein, was present in one patient and was predicted likely pathogenic (**Figure 3E**, **Table 1**).

#### Variants in known POI-associated genes

We also identified mutations in known POI-related genes involved in folliculogenesis and ovarian function, many of which have been validated in mouse models (49).

**Figure 4:**
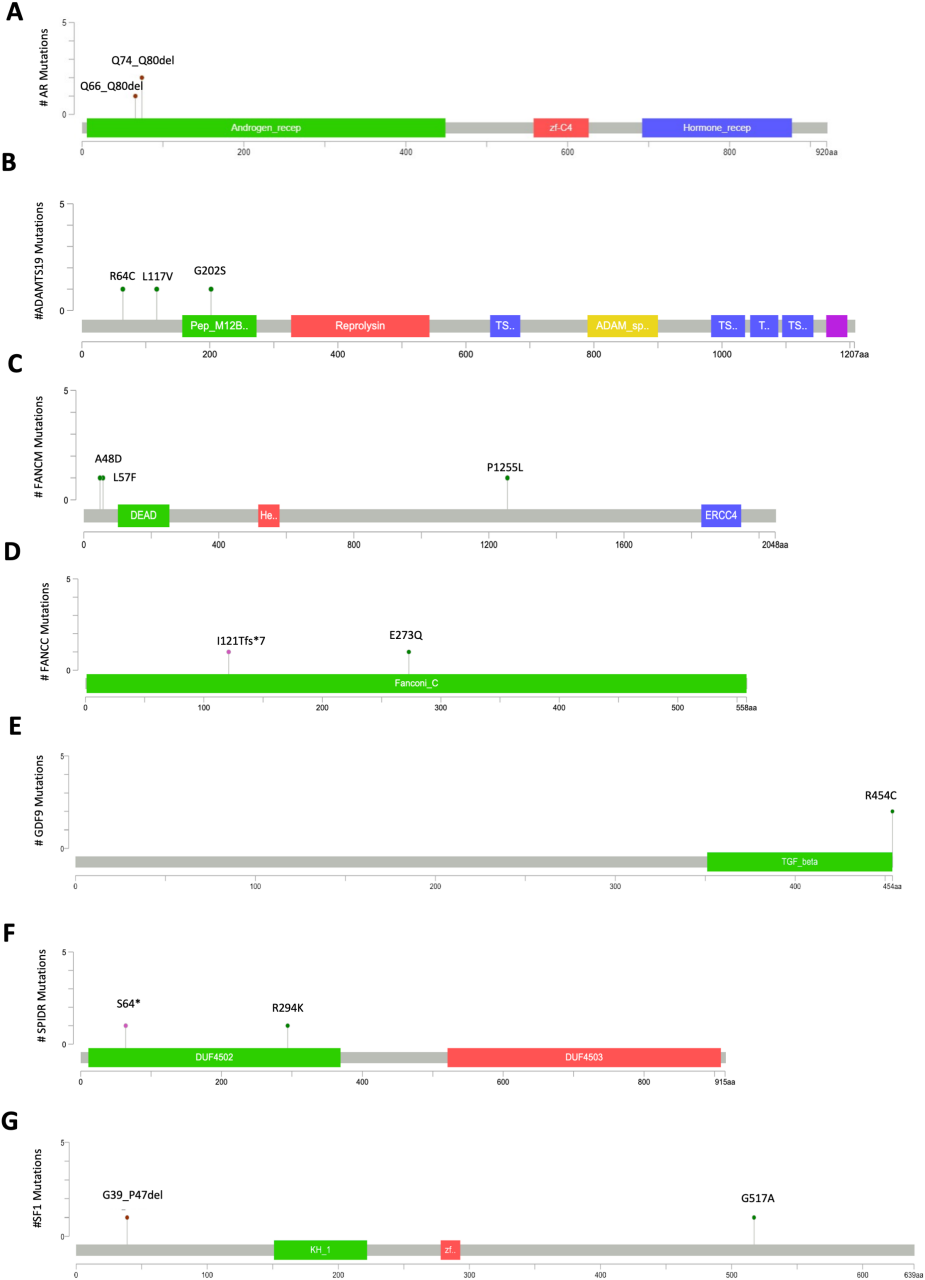
Mutation mapper plots of genes with LP/P variants in nsPOI patients. The plots show the distribution of mutations in *AR***(A)**, *ADAMTS19***(B)**, *FANCM***(C)**, *FANCC***(D)**, *GDF9***(E)**, *SPIDR***(F)**, and *SF1***(G)**. Amino acid changes are indicated above each lollipop, and line length reflects the number of patients carrying the corresponding variant. Plots were generated using the Mutation Mapper tool on the cBioPortal platform (https://www.cbioportal.org/mutation_mapper).

Several X-linked genes are known contributors to POI pathogenesis (50, 51). In addition to PCDH11X, we identified Androgen receptor (AR) mutations in three patients. Two distinct deletions (Q66–80del and Q74–80del) within the poly-Q repeat at the N-terminus were classified as pathogenic (**Figure 4A**, **Supplementary Figure S1**).

Mutations were also found in ADAMTS1 and ADAMTS19, which encode metalloproteinases involved in ovarian extracellular matrix remodeling (52).

Three patients carried R64C, L117V, and G202S missense variants in ADAMTS19 (**Figure 4B**). The R64C mutation, located in the pro-domain crucial for enzyme folding and activity, was deemed likely pathogenic (**Table 1**). Additionally, six patients carried VUS in ADAMTS1, further discussed below.

In recent years, the Fanconi anemia complementation group (*FANC*) genes have also been implicated in POI (22, 23). Mutations were identified in FANCM (3%), FANCC (2%), and FANCG (1%) in our cohort. In FANCM, three missense mutations (A48D, L57F, P1255L) were found (**Figure 4C**, **Table 1**). Although not located in known domains, A48D and L57F were predicted likely pathogenic. FANCC mutations included a potentially pathogenic E273Q substitution and a frameshift mutation (I121Tfs*7) causing a premature stop codon (**Figure 4D**, **Table 1**). One patient carried the W122C mutation in the FANCG protein (**Table 2**). This substitution occurred at an interspecies conserved residue, which given the physicochemical distance between the W and C residues (Grantham score=215) should result in a severe disruption of the protein structure, although it was classified as VUS.

**Table 2:** Mutations in genes carrying only VUS.

N. of patients	Gene	Type of mutation	AA change	gnomAD_NEF	EUR_AF	SIFT	PolyPhen	CADD	Mutation taster	Grantham	ACMG criteria	Classification
1	*ADAMTS1*	missense	A806V	0.01759	NA	0.03	0.581	21.8	B	64	PP3, PM2, PM1	VUS
3	*ADAMTS1*	missense	T732I	0.00012	NA	0.05	0.18	20.7	B	89	PM2, PM1	VUS
2	*ADAMTS1*	missense	T514A	0.00012	0.001	0.01	0	18.49	B	58	PM2	VUS
3	*POF1B*	missense	R315C	0.00074	0.0013	0	1	31	B	180	PM2, PM1	VUS
1	*BNC1*	missense	G661R	NA	NA	0.31	0.007	14.9	B	125	PM2, PP2	VUS
1	*BNC1*	missense	E209K	0.02638	NA	0	1	29.3	D	56	PM2, PM5, PP2	VUS
1	CDKN1B	missense	S7C	NA	NA	0.0	1.0	28.1	B	112	PM2, PP3	VUS
1	*CDKN1B*	missense	P117S	0.06262	NA	0.02	0.167	18.56	D	74	PM2, PS3	VUS
1	*WT1*	missense	S325L	0.008814	NA	0	0.981	32	NA	145	PM2, PM1, PP3	VUS
1	*WT1*	missense	G37S	0.0000398	NA	0.01	0.008	15.6	B	56	PM2, PM1	VUS
1	*MSH4*	missense	S10L	NA	NA	0.31	0	11.6	B	145	PM2	VUS
1	*MSH4*	missense	L35I	0.0003181	0.002	0.03	0.041	8.8	B	5	PM2	VUS
1	*WDR62*	missense	S275L	0.07042	NA	0	1	29.5	B	145	PM2, PP3	VUS
1	*WDR62*	missense	Y336C	0.03519	NA	0.29	1	23.6	B	194	PM2, PP3	VUS
2	BRSK1	missense	P764A	0.005974	0.005	0.22	0	15.03	B	27	PM2, PP2	VUS
1	*FANCG*	missense	W122C	0.0001589	NA	0	1	26.9	B	215	PM2, PM1, PP3	VUS
1	*SOHLH2*	missense	S417L	0.000203	0.001	0	0.947	23	B	145	PM2, PM1, PP3	VUS
1	*SYCE1*	missense	R59Q	0.000229	NA	0.01	1	26.6	B	43	PM2, PM1	VUS
1	*AGTR2*	missense	G21V	0.004099	0.0026	na	na	8.7	B	109	PM2	VUS
1	*FOXO1*	missense	V564M	0.008816	NA	0.46	0.296	22.6	B	21	PM2	VUS
1	*FOXO3*	missense	A140S	0.0001551	NA	0.7	0.052	15.1	B	99	PM2, PM1	VUS
1	BBS9	missense	T865A	0.0005225	0.002	0.06	0.0	15.34	B	58	PM2, BP4	VUS
1	DUSP22	missense	A171T	0.02637	NA	0.85	0.0	11.68	B	58.0	BP4	VUS
1	LARS2	missense	S315L	0.01762	NA	0.64	0.002	20.5	B	145.0	PM2, PM1	VUS
1	NUPR1	missense	R74H	0.0000176	NA	0.18	0.76	23.4	B	29.0	PM2, PM, PP3	VUS
1	POLR3H	missense	V99I	0.02638	NA	0.02	0.544	32	D	29.0	PM2, PP3, PM1	VUS
1	SALL4	missense	G251R	0.008865	NA	0.2	0.661	8.9	B	125.0	PM2, PP2	VUS
1	DMC1	missense	A76T	NA	NA	0.04	0.104	24.6	D	58	PP3, PM2, PM1	VUS

FOR each variant the allele frequency in population databases (GnomAD, 1000 genomes_EUR), the pathogenicity prediction scores (Sift, Polyphen, Mutation Taster, CADD, Grantham) and the final classification according to ACMG criteria are reported. Abbreviations: GnomA NEF, Genome Aggregation Database non-Finnish European; Eur AF, European population Allele Frequency; B, benign; D, deleterious; NA, not available; VUS, variant of uncertain significance.

The gene encoding growth differentiation factor 9 (*GDF9*), crucial for folliculogenesis and oocyte development, was mutated (R454C) in two patients. The R454C mutation occurs at a conserved amino acid position and is likely to have functionally detrimental effects (**Figure 4E**, **Table 1**).

Two patients carried mutations in the Scaffolding Protein Involved in DNA Repair (*SPIDR*) gene, which encodes a scaffold protein involved in HR repair. Both a nonsense mutation (S64*) and a missense mutation (R294K) occurred in the DUF4502 domain, the role of which is not yet fully understood (**Figure 4F**, **Table 1**). The mutation S64* introduces a premature stop codon near the N-terminus and was classified as highly pathogenic.

Two mutations were found in the splicing factor 1 (SF1) gene (**Figure 4G**, **Table 1**). A deletion G39_P47del, in the helix-hairpin domain at the N-terminus of SF1 (53) was classified as LP. This helix-hairpin domain is required for cooperative recognition of 3’ splice sites by stabilizing a unique quaternary arrangement of the SF1-U2AF65 RNA complex during assembly of the spliceosome (54). In contrast, G517A is categorized as VUS in the low complexity region.

Finally, we identified likely pathogenic variants of SOHLH1, AMHR2, TP63, NANOS3, MRPS22, FOXL2 and DMC1 genes in one patient each. These mutations were absent or novel in public databases and had consistently high pathogenicity scores across prediction tools.

Of particular note, we identified a novel Gly323 deletion in SOHLH1, within a low-complexity region involved in folliculogenesis, where other deleterious missense variants have been described (55).

In addition, one patient carries a mutation of the *TP63* gene, which codes for a transcription factor of the p53 family. TP63 is expressed in primordial and primary follicles and preserve the germ line integrity (56). The R487C mutation is located in the linker domain but predicted as potentially deleterious (57, 58).

#### Variants of uncertain significance in functionally relevant genes

**Figure 5:**
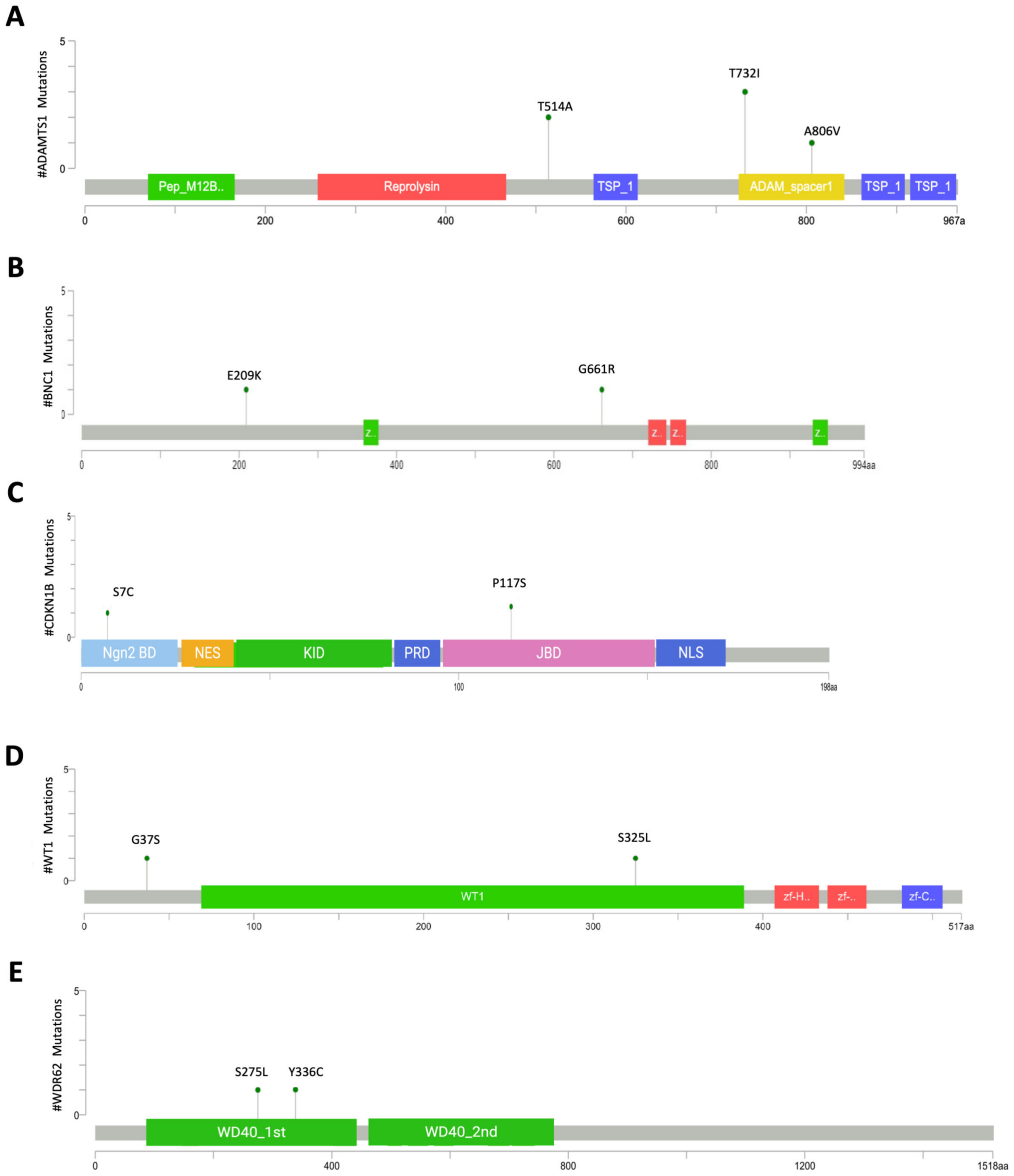
Mutation mapper plots of genes with VUS variants in nsPOI patients. The plots show the distribution of mutations in ADAMTS1 **(A)**, BNC1 **(B)**, CDKN1B **(C)**, WT1 **(D)**, and WDR62 **(E)**. Amino acid changes are indicated above each lollipop, and line length corresponds to the number of patients carrying the respective variant. Plots were generated using the Mutation Mapper tool on the cBioPortal platform (https://www.cbioportal.org/mutation_mapper). CDKN1B and WDR62 plots were modified with Biorender.

Our analysis also identified additional 20 genes that carried only VUS. As previously described, six patients harbored three missense mutations (T514A, T732I, A806V) in another member of the ADAMTS family, ADAMTS1 (**Table 2**). Two patients carried the T514A mutation, located in a linker domain (**Figure 5A**). The T732I variants, present in 3 patients, and the A806V were both located within the ADAM spacer domain (**Figure 5A**). Notably, T732I had the highest Grantham score among the three, indicating that the substitution of threonine—a small, polar, hydrophilic amino acid—with isoleucine—a large, non-polar, hydrophobic residue—could significantly alter protein structure.

Three patients carried the same variant (R315C) in the *POF1B* gene, which has been previously associated with POI (59–61). Although classified as VUS, this variant was predicted to be deleterious by four out of five in silico algorithms (**Table 2**).

The *BNC1* gene, encoding the zinc finger protein Basonuclin 1, is highly expressed in ovarian germ cells and plays a role in transcriptional regulation (62, 63). We identified two missense mutations (E209K and G661R) in the linker domain of the BNC1 protein (**Figure 5B**, **Table 2**). The E209K mutation was predicted to be deleterious by the SIFT, PolyPhen, CADD and MutationTaster algorithms, likely due to the change in charge affecting structural stability and DNA binding capacity.

In the CDKN1B gene, which encodes the cell cycle inhibitor p27^Kip1, two missense variants were identified: S7C, located in the Neurogenin-2 binding domain at the N-terminus (64), and P117S which replaces a highly conserved proline within the Jab1/CSN5 binding domain (**Figure 5C**).

In the WT1 gene, which encodes the WT1 transcription factor, we found two missense mutations (**Figure 5D**). The G37S mutation was located at the N-terminus, which is characterized by a proline- and glutamine-rich DNA-binding domain, while the S325L mutation was located in one of the four zinc finger motifs at the C-terminus (ZnF_CH2H2 domain). Several variants have been described for this gene, including G37S and S325L, both of which are listed with very low frequency in the gnomAD database.

Two patients carried two different mutations, S10L and L35I, in the N-terminus of the protein encoded by the MutS homolog 4 (MSH4) gene, which is involved in the mismatch repair process.

We found two mutations in the gene encoding the WD repeat-containing protein 62 (**Figure 5E**). The mutations S275L and Y336C were located in the first WD40 domain of MABP1/WDR62 (65) of the protein, which is involved in the interaction with Aurora A and binding to microtubules at the spindle pole (66).

In addition, we found VUS mutations in the genes SYCE1, AGTR2, FOXO1, FOXO3, BBS9, DUSP22, LARS2, NUPR1, POLR3H, SALL4 in one patient each. See **Table 2** and **Supplementary File S5**.

### Patients with monogenic or polygenic variants

**Figure 6:**
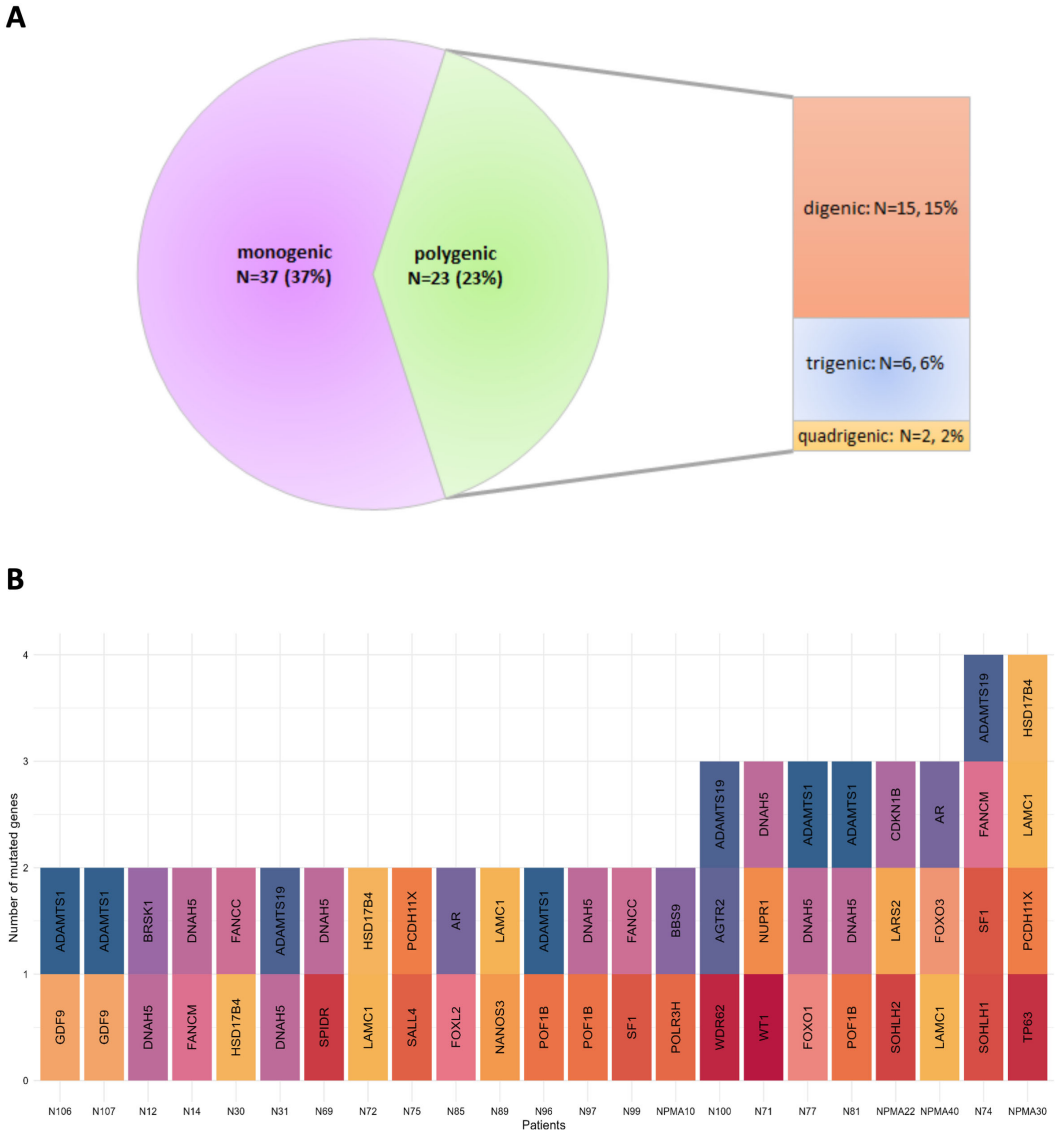
Co-occurrence of mutations in the nsPOI cohort. **(A)** The graph shows the percentage of POI patients in whom one or more genes are simultaneously mutated. **(B)** Stacked bar plot reports the number of mutated genes (y-axis) per patients (x-axis). Gene names are displayed in the bars.

Among the 60 patients with genetic variants, we found that 37 had alterations in a single gene (monogenic patients), while 23 carried variants in two or more genes (polygenic patients) (**Figure 6A**, **Supplementary File S7**). Of the 37 monogenic patients, 27 (73%) carried VUS, while 10 (27%) had likely pathogenic mutations (**Table 3**). One patient carried the truncating pathogenic variant, which occurs in the SPIDR gene.

**Table 3:** The table lists the 37 patients that could be explained by monogenic variants.

Patient_ID	Gene	Chr	Type of mutation	AA change	Classification
N52	*DNAH5*	5	Missense	S3774P	LP
	*DNAH5*	5	Missense	I3568T	VUS
N57	*DNAH5*	5	Missense	Q2949E	VUS
NPMA_4	*DNAH5*	5	Missense	N1420D	VUS
N63	*DNAH5*	5	Missense	L1339R	VUS
N13	*HK3*	5	Missense	P676S	VUS
N120	*HK3*	5	Missense	Q600H	VUS
NPMA_11	*HK3*	5	Missense	Q600H	VUS
N103	*HK3*	5	Missense	I347T	LP
NPMA_7	*HK3*	5	Missense	C237R	LP
N73	*NOTCH2*	1	Missense	I1693T	VUS
N82_v1	*NOTCH2*	1	Missense	R318L	VUS
NPMA_8	*NOTCH2*	1	Missense	A147S	LP
N54	*LAMC1*	1	Missense	R1011H	LP
NPMA_3	*LAMC1*	1	Missense	R1011H	LP
N117	*MSH4*	1	Missense	S10L	VUS
N102	*MSH4*	1	Missense	L35I	VUS
NPMA_12	*PCDH11X*	X	Missense	T790S	VUS
NPMA29	*PCDH11X*	X	Missense	T790S	VUS
N58	*POU5F1*	6	Missense	T116S	VUS
N2	*POU5F1*	6	Missense	P65R	VUS
N118	*BNC1*	15	Missense	G661R	VUS
N62	*BNC1*	15	Missense	E209K	VUS
NPMA14	*HSD17B4*	5	Missense	R658H	VUS
N105	*ADAMTS1*	21	Missense	T732I	VUS
N104	*AMHR2*	12	Missense	G153R	LP
NPMA17	*AR*	X	In_Frame_Del	Q74_Q80del	LP
N113	*FANCG*	9	Missense	W122C	VUS
NPMA_6	*FANCM*	14	Missense	L57F	VUS
NPMA18	*BRSK1*	19	Missense	P764A	VUS
N91	*CDKN1B*	12	Missense	P117S	VUS
NPMA25	*DMC1*	22	Missense	A76T	VUS
N15	*DUSP22*	6	Missense	A171T	VUS
N70	*MRPS22*	3	Missense	V23L	LP
N78	*SPIDR*	8	Nonsense	S64*	P
N92	*SYCE1*	10	Missense	R59Q	VUS
NPMA_49	*WDR62*	19	Missense	Y336C	VUS
N109	*WT1*	11	Missense	G37S	VUS

P, pathogenic; LP, likely pathogenic; VUS, variant of uncertain significance.

Genes exclusively mutated in monogenic patients included HK3, NOTCH2, MSH4, BNC1, POU5F1, DUSP22, FANCG, MRPS22, and SYCE1 (**Table 3**). Four of the 12 patients with DNAH5 mutations had only a VUS mutation of the gene.

Among the polygenic cases, 15 patients had mutations in two genes (15%), 6 patients had mutations in three genes (6%) and only 2 patients had mutations in four genes (2%) (**Figure 6B**, **Supplementary File S7**). Of particular interest, two patients shared the same combination of mutations in the ADAMTS1 (T732I) and GDF9 (R454C) proteins, while two other patients had concurrent but different mutations in the ADAMTS1 and DNAH5 proteins: ADAMTS1-A806V and DNAH5-S2605L; ADAMTS1-T514A and DNAH5-T806I.

### POI genes subjected to CNVs

All POI patients were also screened for germline CNVs using amplicon-based NGS data generated with Ion Reporter software. A pooled DNA sample from patients in the control group served as reference baseline. Based on the MAPD values, 99 out of 100 patients were eligible for CNV analysis (see Materials and Methods). CNVs were detected in 11% of patients, involving SYCE1, DUSP22, and INHBB genes (**Table 4**).

**Table 4:** Chromosome regions with CNVs identified in POI cohort.

Patient	Region	Gene	Type of CNV	CNV length (bp)	Co-occurring mutated genes	Amino acid change	CADD_SV Phred score	StrVCTVRE	X-CNV(MVP)
N38	10q26.3	*SYCE1*	gain	11349			3.558	0.351	0.187
N58	10q26.3	*SYCE1*	gain	11349	*POU5F1*	T116S			
N75	10q26.3	*SYCE1*	gain	11349	*PCDH11X*	T790S			
					*SALL4*	G251R			
N85	10q26.3	*SYCE1*	gain	11349	*AR*	Q66_Q80del			
					*FOXL2*	A234del			
NPMA40	10q26.3	*SYCE1*	gain	11349	*LAMC1*	A1335S			
					*AR*	Q74_Q80del			
					*FOXO3*	A140S			
N47	6p25.3	*DUSP22*	loss	58438			11.06	0.354	0.227
N57	6p25.3	*DUSP22*	gain	58438	*DNAH5*	Q2949E	13.79	0.264	0.154
N90	6p25.3	*DUSP22*	gain	58438					
NPMA_3	6p25.3	*DUSP22*	gain	58438	*LAMC1*	R1011H			
NPMA_7	6p25.3	*DUSP22*	gain	58438	*HK3*	C237R			
N61	2q14.2	*INHBB*	loss	3890			13.86	0.671	0.700

The table shows CNVs and co-occurred mutations in genes previously associated to POI. For each CNV chromosome region, type of CNV, genomic coordinates CNV length, gene, CADD SV Phred, StrVCTVRE, X-CNV(MVP), co-occurred mutated genes and their amino acid changes scores are indicated.

Five patients had CN amplifications of the SYCE1 gene on chromosome 10q26.3 and all were identified as focal (<3Mb). Four of the five patients with copy number gains also had missense mutations and deletions in other genes (**Table 4**). Notably, one patient with SYCE1 CN gains also had LP variants in AR (Q66_Q80del) and FOXL2 (A234del) (**Table 4**), while the other had LP mutations of LAMC1 (A1335S) and AR (Q74_Q80del) and a VUS (A140S) in the FOXO3 gene.

Five patients had focal CNVs of the DUSP22 gene on chromosome 6p25. Four patients had CN gains of the gene, while one patient had a copy number loss of the gene. Three of the four patients with copy number gain also had a pathogenic mutation each in DNAH5 (Q2949E), LAMC1 (R1011H) and HK3 (C237R) (**Table 3**).

We also found a copy number loss of the INHBB gene on chromosome 2q14.2 in a patient who had no mutations in other genes (**Table 3**).

For pathogenetic classification of the identified CNV, we used the StrVCTVRE, MVP and CADD_SV_Phred scores (see Material and Methods for more details). According to the scores obtained, the microdeletion found in the INHBB gene was classified as likely pathogenic. **Supplementary Figure S2** shows representative Q-PCR analysis of CNVs in SYCE1, DUSP22 and INHBB genes.

## Discussion

In this study, we analyzed a cohort of 100 Italian women affected by non-syndromic primary ovarian insufficiency (nsPOI), selected using the OvAge^©^ algorithm (35), to investigate the contribution of genetic alterations using a targeted next-generation sequencing (NGS) approach. Preliminary validation has shown that OvAge discriminates pathological conditions such as POI and Polycystic Ovary Syndrome (PCOS), which are often misclassified by individual tests (35). In longitudinal observations, OvAge was also able to anticipate menopause within 1–2 years in women predicted to have an ovarian age close to 50 years, suggesting potential prognostic value. However, it was derived from a single, albeit large, population and requires advanced ultrasound technology, so external validation, broader clinical application, and integration with genetic information are warranted.

In the present study, by integrating sequencing data with a panel of 72 POI-related genes, we identified 78 rare variants distributed across 41 genes, which were absent in 200 matched controls. Variants were classified following ACMG guidelines, resulting in a diagnostic yield of 60%, with 23% of patients carrying pathogenic or likely pathogenic (LP/P) variants and 37% harboring only variants of uncertain significance (VUS).

Our findings further support the notion that nsPOI is genetically heterogeneous disorder, with both monogenic and polygenic contributions. Notably, DNAH5 emerged as the most frequently mutated gene (12%), with several variants located in conserved motor domains.

DNAH5 encodes a microtubule-associated motor protein involved in ciliary motility. Mutations in DNAH5 have previously been linked to primary ciliary dyskinesia (PCD) (67), as well as non-syndromic asthenozoospermia and hypospadias (68, 69). Aboura and colleagues identified chromosomal amplifications at 5p14.3, the locus of *DNAH5*, in a POI cohort. To our knowledge, this is the first report describing DNAH5 mutations in nsPOI patients.

We also identified mutations in the gene encoding the laminin subunit gamma-1 (*LAMC1*). LAMC1 is known to interact with other laminin family proteins in the extracellular matrix to promote ovarian follicle development (70, 71). Overexpression of LAMC1 is involved in the progression of gynecologic cancers (72, 73) and predicts poor prognosis in gastric and esophageal cancers (74–77). In contrast, only one missense mutation (78) and one SNP haplotype (79) have been associated with increased POI risk without functional characterization. In this work, we identified four novel missense mutations (R1011H, Y1035S, A1239V, A1335S) in *LAMC1* that are very rare or absent in the general population. In particular, the mutation R1011H, which is localized in the EGF_like domain and occurred in three different patients, was classified as likely pathogenic.

Our data also highlight the relevance of the ADAMTS family in ovarian function (80, 81). We detected pathogenic or VUS variants in both ADAMTS1 and ADAMTS19, proteases known to regulate folliculogenesis and ovulation via extracellular matrix remodeling (82–84).

The role of ADAMTS1 and ADAMTS19 in reproductive function has been demonstrated using mouse models: *ADAMTS1* homozygous knock-out mice had fewer ovarian follicles (85), while the protease *ADAMTS19* is overexpressed in the gonads of female mice (86). Regarding the role of ADAMTS19 in nsPOI, so far only SNPs in intronic regions of *ADAMTS19* have been potentially associated with POI in both Caucasian (87) and Asian populations (79). In our study, we described three novel mutations in *ADAMTS1* (A806V, T732I and T514A) found in six patients. In particular, the mutation T732I, which occurred in three patients, is located in the spacer domain, which is necessary for association with ECM components and regulation of enzyme activity (88). In addition, we identified three missense mutations (R64C, L117V, G202S) of *ADAMTS1*9, of which the R64C mutation was classified as likely pathogenic according to the ACMG criteria.

The *HSD17B4* gene, also known as D-bifunctional protein (DBP), is a bifunctional enzyme involved in the conversion of androstenedione to testosterone and estrone to estradiol. In this study, we found four different missense mutations (I53M, C214S, R658H and A741S) in *HSD17B4* gene in 4 different patients. Studies on Perrault syndrome (89) provided evidence of the importance of HSD174B for healthy ovarian function. In addition, Puyan et al. found a haplotype and two missense SNPs in the HSD17B4 gene associated with susceptibility to POF in a genetic case-control association study (79).

Remarkably, 31% of mutated genes in our study were involved in DNA repair and meiosis, pathways essential for oocyte integrity. We found mutations in three different genes of the FANC family, FANCM, FANCC and FANCG, whose role in repair during HR in meiosis has been recently investigated (90). Our results support the observations that defects in the FANC genes can impair normal oogenesis. In our cohort, 3 of the five mutations identified in the FANCM and FANCC genes were classified as LP or P (**Table 2**). In addition, we found mutations in the gene MSH4, which is required for optimal reciprocal recombination and appropriate segregation of homologous chromosomes during meiosis I (91). Mutations of this gene have recently been associated with failure of gametogenesis in both sexes (92). A causal role may also be attributed to mutations in the homologous recombination repair gene SPIDR gene, whose alteration has been associated with gonadal disgenesia (93) and ovarian failure (23).

We have also identified novel variants in genes previously associated with POI such as PCDH11X, AR, TP63, BNC1, WT1, CDKN1B, SYCE1 as well as previously described mutations such as R454C in GDF9, which is considered one of the causative alterations in POI (94). It is noteworthy that, to our knowledge, no POI-associated missense mutations for PCDH11X have been reported to date, only CNV (52). In addition, three patients had two different deletions in the poly-Q region of the AR gene. Previous reports have described (95–97) only point mutations of the gene encoding the AR in POI patients, while short poly-Q polymorphisms have been associated with poorer prognosis only in endometrial cancer (98).

Recent data have identified TP63 mutations in syndromic and non-syndromic POI, associated with oocyte apoptosis and early ovarian reserve depletion (17, 61, 99). The R487C mutation identified in our study has previously been linked to colorectal cancer (57), low-grade gliomas (58), and more recently, to pyroptosis-related gene networks (58, 100, 101). This supports a possible role for p63 as a high-risk biomarker in cancer and reproductive disorders.

Interestingly, the P117S mutation in the cell cycle inhibitor CDKN1B has been associated with multiple endocrine neoplasia type IV (MEN4) (102, 103). This mutation is located in the domain of binding to Jab1/CSN5, which promotes the translocation of p27 from the nucleus to the cytoplasm, thereby favoring cell proliferation (104).

POI-associated genes include the Synaptonemal Complex Central Element 1 (SYCE1) gene which encodes a member of the synaptonemal complex that links homologous chromosomes during prophase I of meiosis. Allelic variants of this gene have been associated with premature ovarian failure (105) and spermatogenic failure. We found a VUS in this gene (R59K) that was reported as a somatic mutation in a patient with malignant skin cancer (106).

Of particular interest, several variants were shared among multiple patients, and distinct mutations in the same gene were observed across the cohort. In 37 patients, POI could be attributed to monogenic variants, while in 23, mutations in two or more genes suggested a polygenic etiology. We found that two patients had a specific combination of mutations in the ADAMTS1 (T732I) and GDF9 (R454C) genes. Two patients carried both DNAH5 variants, S3774P and I3568T. As parental DNA was unavailable, phasing could not be established, and compound heterozygosity for an autosomal recessive mechanism remains unconfirmed. Segregation studies in relatives and analysis of a larger cohort are planned to assess the contribution of this co-mutation to nsPOI.

CNV analysis identified alterations in *SYCE1, DUSP22*, and *INHBB*. Of these, only the INHBB CNV was classified as likely pathogenic by prediction tools. Although SNV mutations in the INHBB gene were reported in a previous study (107), no evidence of POI-causing CNV in this gene has been described to date. *INHBB* encodes the βB subunit of inhibin/activin dimers which regulate granulosa cell proliferation and folliculogenesis (108, 109). Loss of INHBB function may impair activin-mediated signaling, leading to defective follicular maturation and contributing to premature ovarian failure. We speculate that CNVs involving DUSP22 may dysregulate JNK signaling (110), thereby promoting apoptosis during follicle maturation. Collectively, these findings suggest that CNVs may play a significant role in the pathogenesis of nsPOI. From a clinical perspective, identification of pathogenic variants—including both SNVs and CNVs—offers opportunities for translation into patient care. Preimplantation genetic testing (PGT) could help prevent transmission of deleterious variants in families with a history of POI. Moreover, women at increased risk could be counseled regarding early fertility preservation strategies, such as oocyte cryopreservation. As the disrupted signaling pathways become more clearly defined, these insights may also pave the way for targeted therapeutic approaches, including pharmacological modulation and, in the future, gene-based interventions.

In summary, we employed a customized targeted NGS panel to analyze DNA from 100 POI patients and 200 controls. The approach yielded a diagnostic rate of 60%, implicating 42 genes. These findings provide new insights into the complex genetic architecture of POI.

We acknowledge that the pathogenic potential of many variants identified requires confirmation through functional validation in cell and animal models. Several VUS in our cohort appear to be strong candidates for reclassification as “likely pathogenic.” Consistent with recent reports, a substantial proportion of VUS are ultimately reclassified, although the average time to reclassification is approximately 2–3 years, underscoring the need for periodic reanalysis (111–113).

Notably, variants such as BNC1 E209K, CDKN1B P117S, WT1 S325L, WDR62 S275L, FANCG W122C, SOHLH2 S147L, and SYCE R59K showed high deleteriousness scores, were located in conserved domains, and were absent or extremely rare in population databases. However, additional functional or segregation evidence will be required to support their reinterpretation.

Expanding the cohort size may further improve sensitivity and strengthen genetic associations. We are acknowledge the absence of segregation testing in family members, which could have clarified the significance of several VUS. This was primarily due to budgetary constraints, as the project was supported by a ministerial grant that covered only affected patients and did not extend to relatives. Despite these limitations, the integration of clinical, hormonal, and ultrasonographic data (via the OvAge algorithm) with targeted NGS represents a promising strategy for early POI diagnosis and personalized management. While available treatments can assist with fertility, the irreversible depletion of ovarian reserve in POI remains incurable. As affected women are also at increased risk for comorbidities that reduce life expectancy, early identification of genetic risk could improve long-term outcomes and quality of life.

## Data Availability

The original contributions presented in the study are included in the article/**Supplementary Material**. Further inquiries can be directed to the corresponding authors.

## Supplementary material

Supplementary Figure 1 Sanger sequencing analysis. Sanger sequencing was used to confirm the indicated mutations of *DNAH5***(A)**, *GDF9***(B)**, *LAMC1***(C, D)**, and *AR***(E)** genes.

Supplementary Figure 2 Q-RT-PCR analysis. The graphs show the CN gains of *SYCE1***(A)** and *DUSP22***(B)** and the CN losses of *INHBB***(C)** in each of the indicated patients. Values are expressed as CN ratios compared to a pool of control samples arbitrarily set as a reference for the diploid state (CN = 2). Statistical significance was calculated in relation to the pool of control samples: *p<0.05, **p<0.01.

## References

[1] Smith S Pfeifer SM Collins JA . Diagnosis and management of female infertility. Jama. (2003) 290:1767–70. doi: 10.1001/jama.290.13.1767, PMID: 14519712

[2] De Vos M Devroey P Fauser BC . Primary ovarian insufficiency. Lancet. (2010) 376:911–21. doi: 10.1016/s0140-6736(10)60355-8, PMID: 20708256

[3] Fortuño C Labarta E . Genetics of primary ovarian insufficiency: A review. J Assist Reprod Genet. (2014) 31:1573–85. doi: 10.1007/s10815-014-0342-9, PMID: 25227694 PMC4250468

[4] Nelson LM . Clinical practice. Primary ovarian insufficiency. N Engl J Med. (2009) 360:606–14. doi: 10.1056/NEJMcp0808697, PMID: 19196677 PMC2762081

[5] Maclaran K Panay N . Current concepts in premature ovarian insufficiency. Womens Health (Lond). (2015) 11:169–82. doi: 10.2217/whe.14.82, PMID: 25776291

[6] Podfigurna-Stopa A Czyzyk A Grymowicz M Smolarczyk R Katulski K Czajkowski K . Premature ovarian insufficiency: the context of long-term effects. J Endocrinol Invest. (2016) 39:983–90. doi: 10.1007/s40618-016-0467-z, PMID: 27091671 PMC4987394

[7] Jiao X Zhang H Ke H Zhang J Cheng L Liu Y . Premature ovarian insufficiency: phenotypic characterization within different etiologies. J Clin Endocrinol Metab. (2017) 102:2281–90. doi: 10.1210/jc.2016-3960, PMID: 28368522

[8] Rudnicka E Kruszewska J Klicka K Kowalczyk J Grymowicz M Skórska J . Premature ovarian insufficiency - aetiopathology, epidemiology, and diagnostic evaluation. Prz Menopauzalny. (2018) 17:105–8. doi: 10.5114/pm.2018.78550, PMID: 30357004 PMC6196779

[9] Sharif K Watad A Bridgewood C Kanduc D Amital H Shoenfeld Y . Insights into the autoimmune aspect of premature ovarian insufficiency. Best Pract Res Clin Endocrinol Metab. (2019) 33:101323. doi: 10.1016/j.beem.2019.101323, PMID: 31606343

[10] Szeliga A Calik-Ksepka A Maciejewska-Jeske M Grymowicz M Smolarczyk K Kostrzak A . Autoimmune diseases in patients with premature ovarian insufficiency-our current state of knowledge. Int J Mol Sci. (2021) 22. doi: 10.3390/ijms22052594, PMID: 33807517 PMC7961833

[11] Panay N Kalu E . Management of premature ovarian failure. Best Pract Res Clin Obstet Gynaecol. (2009) 23:129–40. doi: 10.1016/j.bpobgyn.2008.10.008, PMID: 19091633

[12] Chapman C Cree L Shelling AN . The genetics of premature ovarian failure: current perspectives. Int J Womens Health. (2015) 7:799–810. doi: 10.2147/ijwh.S64024, PMID: 26445561 PMC4590549

[13] Kirshenbaum M Orvieto R . Premature ovarian insufficiency (Poi) and autoimmunity-an update appraisal. J Assist Reprod Genet. (2019) 36:2207–15. doi: 10.1007/s10815-019-01572-0, PMID: 31440958 PMC6885484

[14] Ke H Tang S Guo T Hou D Jiao X Li S . Landscape of pathogenic mutations in premature ovarian insufficiency. Nat Med. (2023) 29:483–92. doi: 10.1038/s41591-022-02194-3, PMID: 36732629 PMC9941050

[15] Yang Q Mumusoglu S Qin Y Sun Y Hsueh AJ . A kaleidoscopic view of ovarian genes associated with premature ovarian insufficiency and senescence. FASEB J. (2021) 35:e21753. doi: 10.1096/fj.202100756R, PMID: 34233068

[16] Aboura A Dupas C Tachdjian G Portnoï MF Bourcigaux N Dewailly D . Array comparative genomic hybridization profiling analysis reveals deoxyribonucleic acid copy number variations associated with premature ovarian failure. J Clin Endocrinol Metab. (2009) 94:4540–6. doi: 10.1210/jc.2009-0186, PMID: 19837940

[17] Bestetti I Castronovo C Sironi A Caslini C Sala C Rossetti R . High-resolution array-cgh analysis on 46,Xx patients affected by early onset primary ovarian insufficiency discloses new genes involved in ovarian function. Hum Reprod. (2019) 34:574–83. doi: 10.1093/humrep/dey389, PMID: 30689869 PMC6389867

[18] Bouilly J Beau I Barraud S Bernard V Azibi K Fagart J . Identification of multiple gene mutations accounts for a new genetic architecture of primary ovarian insufficiency. J Clin Endocrinol Metab. (2016) 101:4541–50. doi: 10.1210/jc.2016-2152, PMID: 27603904

[19] Desai S Wood-Trageser M Matic J Chipkin J Jiang H Bachelot A . Mcm8 and mcm9 nucleotide variants in women with primary ovarian insufficiency. J Clin Endocrinol Metab. (2017) 102:576–82. doi: 10.1210/jc.2016-2565, PMID: 27802094 PMC5413161

[20] Eskenazi S Bachelot A Hugon-Rodin J Plu-Bureau G Gompel A Catteau-Jonard S . Next generation sequencing should be proposed to every woman with "Idiopathic" Primary ovarian insufficiency. J Endocr Soc. (2021) 5:bvab032. doi: 10.1210/jendso/bvab032, PMID: 34095689 PMC8169040

[21] Fonseca DJ Patiño LC Suárez YC de Jesús Rodríguez A Mateus HE Jiménez KM . Next generation sequencing in women affected by nonsyndromic premature ovarian failure displays new potential causative genes and mutations. Fertil Steril. (2015) 104:154–62.e2. doi: 10.1016/j.fertnstert.2015.04.016, PMID: 25989972

[22] França MM Mendonca BB . Genetics of ovarian insufficiency and defects of folliculogenesis. Best Pract Res Clin Endocrinol Metab. (2022) 36:101594. doi: 10.1016/j.beem.2021.101594, PMID: 34794894

[23] Heddar A Ogur C Da Costa S Braham I Billaud-Rist L Findikli N . Genetic landscape of a large cohort of primary ovarian insufficiency: new genes and pathways and implications for personalized medicine. eBioMedicine. (2022) 84. doi: 10.1016/j.ebiom.2022.104246, PMID: 36099812 PMC9475279

[24] Jaillard S Bell K Akloul L Walton K McElreavy K Stocker WA . New insights into the genetic basis of premature ovarian insufficiency: novel causative variants and candidate genes revealed by genomic sequencing. Maturitas. (2020) 141:9–19. doi: 10.1016/j.maturitas.2020.06.004, PMID: 33036707

[25] Laissue P Vinci G Veitia RA Fellous M . Recent advances in the study of genes involved in non-syndromic premature ovarian failure. Mol Cell Endocrinol. (2008) 282:101–11. doi: 10.1016/j.mce.2007.11.005, PMID: 18164539

[26] McGuire MM Bowden W Engel NJ Ahn HW Kovanci E Rajkovic A . Genomic analysis using high-resolution single-nucleotide polymorphism arrays reveals novel microdeletions associated with premature ovarian failure. Fertil Steril. (2011) 95:1595–600. doi: 10.1016/j.fertnstert.2010.12.052, PMID: 21256485 PMC3062633

[27] Qin Y Jiao X Simpson JL Chen ZJ . Genetics of primary ovarian insufficiency: new developments and opportunities. Hum Reprod Update. (2015) 21:787–808. doi: 10.1093/humupd/dmv036, PMID: 26243799 PMC4594617

[28] Tšuiko O Nõukas M Žilina O Hensen K Tapanainen JS Mägi R . Copy number variation analysis detects novel candidate genes involved in follicular growth and oocyte maturation in a cohort of premature ovarian failure cases. Hum Reprod. (2016) 31:1913–25. doi: 10.1093/humrep/dew142, PMID: 27301361 PMC4974666

[29] Illés A Pikó H Árvai K Donka V Szepesi O Kósa J . Screening of premature ovarian insufficiency associated genes in hungarian patients with next generation sequencing. BMC Med Genomics. (2024) 17:98. doi: 10.1186/s12920-024-01873-z, PMID: 38649916 PMC11036647

[30] Jolly A Bayram Y Turan S Aycan Z Tos T Abali ZY . Exome sequencing of a primary ovarian insufficiency cohort reveals common molecular etiologies for a spectrum of disease. J Clin Endocrinol Metab. (2019) 104:3049–67. doi: 10.1210/jc.2019-00248, PMID: 31042289 PMC6563799

[31] Liu P Zhang X Hu J Cui L Zhao S Jiao X . Dysregulated cytokine profile associated with biochemical premature ovarian insufficiency. Am J Reprod Immunol. (2020) 84:e13292. doi: 10.1111/aji.13292, PMID: 32564444 PMC7539985

[32] Luo W Ke H Tang S Jiao X Li Z Zhao S . Next-generation sequencing of 500 poi patients identified novel responsible monogenic and oligogenic variants. J Ovarian Res. (2023) 16:39. doi: 10.1186/s13048-023-01104-6, PMID: 36793102 PMC9930292

[33] Vogt EC Bratland E Berland S Berentsen R Lund A Björnsdottir S . Improving diagnostic precision in primary ovarian insufficiency using comprehensive genetic and autoantibody testing. Hum Reprod. (2024) 39:177–89. doi: 10.1093/humrep/dead233, PMID: 37953503 PMC10767963

[34] Venturella R De Vivo V Carlea A D'Alessandro P Saccone G Arduino B . The genetics of non-syndromic primary ovarian insufficiency: A systematic review. Int J Fertil Steril. (2019) 13:161–8. doi: 10.22074/ijfs.2019.5599, PMID: 31310068 PMC6642427

[35] Venturella R Lico D Sarica A Falbo MP Gulletta E Morelli M . Ovage: A new methodology to quantify ovarian reserve combining clinical, biochemical and 3d-ultrasonographic parameters. J Ovarian Res. (2015) 8:21. doi: 10.1186/s13048-015-0149-z, PMID: 25881987 PMC4392473

[36] Flanagan SE Patch AM Ellard S . Using sift and polyphen to predict loss-of-function and gain-of-function mutations. Genet Test Mol Biomarkers. (2010) 14:533–7. doi: 10.1089/gtmb.2010.0036, PMID: 20642364

[37] Rentzsch P Schubach M Shendure J Kircher M . Cadd-splice-improving genome-wide variant effect prediction using deep learning-derived splice scores. Genome Med. (2021) 13:31. doi: 10.1186/s13073-021-00835-9, PMID: 33618777 PMC7901104

[38] Grantham R . Amino acid difference formula to help explain protein evolution. Science. (1974) 185:862–4. doi: 10.1126/science.185.4154.862, PMID: 4843792

[39] Schwarz JM Rödelsperger C Schuelke M Seelow D . Mutationtaster evaluates disease-causing potential of sequence alterations. Nat Methods. (2010) 7:575–6. doi: 10.1038/nmeth0810-575, PMID: 20676075

[40] Li Q Wang K . Intervar: clinical interpretation of genetic variants by the 2015 acmg-amp guidelines. Am J Hum Genet. (2017) 100:267–80. doi: 10.1016/j.ajhg.2017.01.004, PMID: 28132688 PMC5294755

[41] Richards S Aziz N Bale S Bick D Das S Gastier-Foster J . Standards and guidelines for the interpretation of sequence variants: A joint consensus recommendation of the american college of medical genetics and genomics and the association for molecular pathology. Genet Med. (2015) 17:405–24. doi: 10.1038/gim.2015.30, PMID: 25741868 PMC4544753

[42] Lin P Hartz SM Wang JC Krueger RF Foroud TM Edenberg HJ . Copy number variation accuracy in genome-wide association studies. Hum Hered. (2011) 71:141–7. doi: 10.1159/000324683, PMID: 21778733 PMC3153341

[43] Bignell GR Greenman CD Davies H Butler AP Edkins S Andrews JM . Signatures of mutation and selection in the cancer genome. Nature. (2010) 463:893–8. doi: 10.1038/nature08768, PMID: 20164919 PMC3145113

[44] Kleinert P Kircher M . A framework to score the effects of structural variants in health and disease. Genome Res. (2022) 32:766–77. doi: 10.1101/gr.275995.121, PMID: 35197310 PMC8997355

[45] Sharo AG Hu Z Sunyaev SR Brenner SE . Strvctvre: A supervised learning method to predict the pathogenicity of human genome structural variants. Am J Hum Genet. (2022) 109:195–209. doi: 10.1016/j.ajhg.2021.12.007, PMID: 35032432 PMC8874149

[46] Zhang L Shi J Ouyang J Zhang R Tao Y Yuan D . X-cnv: genome-wide prediction of the pathogenicity of copy number variations. Genome Med. (2021) 13:132. doi: 10.1186/s13073-021-00945-4, PMID: 34407882 PMC8375180

[47] De Marco C Zoppoli P Rinaldo N Morganella S Morello M Zuccalà V . Genome-wide analysis of copy number alterations led to the characterisation of pdcd10 as oncogene in ovarian cancer. Transl Oncol. (2021) 14:101013. doi: 10.1016/j.tranon.2021.101013, PMID: 33516089 PMC7846933

[48] Schmittgen TD Livak KJ . Analyzing real-time pcr data by the comparative C(T) method. Nat Protoc. (2008) 3:1101–8. doi: 10.1038/nprot.2008.73, PMID: 18546601

[49] Sullivan SD Castrillon DH . Insights into Primary Ovarian Insufficiency through Genetically Engineered Mouse Models. Semin Reprod Med. (2011) 29:283–98. doi: 10.1055/s-0031-1280914, PMID: 21972066

[50] Persani L Rossetti R Cacciatore C Bonomi M . Primary ovarian insufficiency: X chromosome defects and autoimmunity. J Autoimmun. (2009) 33:35–41. doi: 10.1016/j.jaut.2009.03.004, PMID: 19346101

[51] Yang X Yang L . Current understanding of the genomic abnormities in premature ovarian failure: chance for early diagnosis and management. Front Med (Lausanne). (2023) 10:1194865. doi: 10.3389/fmed.2023.1194865, PMID: 37332766 PMC10274511

[52] Knauff EA Blauw HM Pearson PL Kok K Wijmenga C Veldink JH . Copy number variants on the X chromosome in women with primary ovarian insufficiency. Fertil Steril. (2011) 95:1584–8.e1. doi: 10.1016/j.fertnstert.2011.01.018, PMID: 21316664

[53] UniProt . Splicing factor 1-interpro classification of protein families (2025). Available online at: https://www.ebi.ac.uk/interpro/protein/UniProt/Q15637 (Accessed March 12, 2025).

[54] Zhang Y Madl T Bagdiul I Kern T Kang HS Zou P . Structure, phosphorylation and U2af65 binding of the N-terminal domain of splicing factor 1 during 3'-splice site recognition. Nucleic Acids Res. (2013) 41:1343–54. doi: 10.1093/nar/gks1097, PMID: 23175611 PMC3553976

[55] Zhao S Li G Dalgleish R Vujovic S Jiao X Li J . Transcription factor sohlh1 potentially associated with primary ovarian insufficiency. Fertil Steril. (2015) 103:548–53.e5. doi: 10.1016/j.fertnstert.2014.11.011, PMID: 25527234

[56] Suh EK Yang A Kettenbach A Bamberger C Michaelis AH Zhu Z . P63 protects the female germ line during meiotic arrest. Nature. (2006) 444:624–8. doi: 10.1038/nature05337, PMID: 17122775

[57] Quintana I Mur P Terradas M García-Mulero S Aiza G Navarro M . Potential involvement of nsd1, krt24 and acaca in the genetic predisposition to colorectal cancer. Cancers (Basel). (2022) 14. doi: 10.3390/cancers14030699, PMID: 35158968 PMC8833793

[58] Wang H Yan L Liu L Lu X Chen Y Zhang Q . A pyroptosis gene-based prognostic model for predicting survival in low-grade glioma. PeerJ. (2023) 11:e16412. doi: 10.7717/peerj.16412, PMID: 38025749 PMC10652862

[59] Lacombe A Lee H Zahed L Choucair M Muller JM Nelson SF . Disruption of pof1b binding to nonmuscle actin filaments is associated with premature ovarian failure. Am J Hum Genet. (2006) 79:113–9. doi: 10.1086/505406, PMID: 16773570 PMC1474115

[60] Ledig S Preisler-Adams S Morlot S Liehr T Wieacker P . Premature ovarian failure caused by a heterozygous missense mutation in pof1b and a reciprocal translocation 46,X,T(X;3)(Q21.1;Q21.3). Sex Dev. (2015) 9:86–90. doi: 10.1159/000373906, PMID: 25676666

[61] Tucker EJ Tan TY Stark Z Sinclair AH . Genomic testing in premature ovarian insufficiency: proceed with caution. Biol Reprod. (2022) 107:1155–8. doi: 10.1093/biolre/ioac153, PMID: 35908231

[62] Iuchi S Green H . Nuclear localization of basonuclin in human keratinocytes and the role of phosphorylation. Proc Natl Acad Sci U.S.A. (1997) 94:7948–53. doi: 10.1073/pnas.94.15.7948, PMID: 9223293 PMC21535

[63] Tian Q Kopf GS Brown RS Tseng H . Function of basonuclin in increasing transcription of the ribosomal rna genes during mouse oogenesis. Development. (2001) 128:407–16. doi: 10.1242/dev.128.3.407, PMID: 11152639

[64] Saida K Fukuda T Scott DA Sengoku T Ogata K Nicosia A . Otud5 variants associated with X-linked intellectual disability and congenital malformation. Front Cell Dev Biol. (2021) 9:631428. doi: 10.3389/fcell.2021.631428, PMID: 33748114 PMC7965969

[65] UniProt . Wd repeat-containing protein 62 - interpro classification of protein families. (2025). Available online at: https://www.ebi.ac.uk/interpro/protein/UniProt/O43379/ (Accessed March 17, 2025).

[66] Huang J Liang Z Guan C Hua S Jiang K . Wdr62 regulates spindle dynamics as an adaptor protein between tpx2/aurora a and katanin. J Cell Biol. (2021) 220. doi: 10.1083/jcb.202007167, PMID: 34137789 PMC8240853

[67] Olbrich H Häffner K Kispert A Völkel A Volz A Sasmaz G . Mutations in dnah5 cause primary ciliary dyskinesia and randomization of left-right asymmetry. Nat Genet. (2002) 30:143–4. doi: 10.1038/ng817, PMID: 11788826

[68] Chen Z Lei Y Finnell RH Ding Y Su Z Wang Y . Whole-exome sequencing study of hypospadias. iScience. (2023) 26:106663. doi: 10.1016/j.isci.2023.106663, PMID: 37168556 PMC10165268

[69] Zuccarello D Ferlin A Cazzadore C Pepe A Garolla A Moretti A . Mutations in dynein genes in patients affected by isolated non-syndromic asthenozoospermia. Hum Reprod. (2008) 23:1957–62. doi: 10.1093/humrep/den193, PMID: 18492703

[70] Irving-Rodgers HF Rodgers RJ . Extracellular matrix of the developing ovarian follicle. Semin Reprod Med. (2006) 24:195–203. doi: 10.1055/s-2006-948549, PMID: 16944417

[71] Rodgers RJ Irving-Rodgers HF Russell DL . Extracellular matrix of the developing ovarian follicle. Reproduction. (2003) 126:415–24. doi: 10.1530/rep.0.1260415, PMID: 14525524

[72] Kashima H Wu RC Wang Y Sinno AK Miyamoto T Shiozawa T . Laminin C1 expression by uterine carcinoma cells is associated with tumor progression. Gynecol Oncol. (2015) 139:338–44. doi: 10.1016/j.ygyno.2015.08.025, PMID: 26343160 PMC4862403

[73] Kunitomi H Kobayashi Y Wu RC Takeda T Tominaga E Banno K . Lamc1 is a prognostic factor and a potential therapeutic target in endometrial cancer. J Gynecol Oncol. (2020) 31:e11. doi: 10.3802/jgo.2020.31.e11, PMID: 31912669 PMC7044014

[74] Diao B Yang P . Comprehensive analysis of the expression and prognosis for laminin genes in ovarian cancer. Pathol Oncol Res. (2021) 27:1609855. doi: 10.3389/pore.2021.1609855, PMID: 34512203 PMC8423899

[75] Fang L Che Y Zhang C Huang J Lei Y Lu Z . Lamc1 upregulation via tgfβ Induces inflammatory cancer-associated fibroblasts in esophageal squamous cell carcinoma via nf-Kb-cxcl1-stat3. Mol Oncol. (2021) 15:3125–46. doi: 10.1002/1878-0261.13053, PMID: 34218518 PMC8564640

[76] Fang Y Dou R Huang S Han L Fu H Yang C . Lamc1-mediated preadipocytes differentiation promoted peritoneum pre-metastatic niche formation and gastric cancer metastasis. Int J Biol Sci. (2022) 18:3082–101. doi: 10.7150/ijbs.70524, PMID: 35541892 PMC9066104

[77] Han ZR Jiang XL Fan WC . Lamc1 is related to the poor prognosis of patients with gastric cancer and facilitates cancer cell Malignancies. Neoplasma. (2021) 68:711–8. doi: 10.4149/neo_2021_201117N1239, PMID: 33884884

[78] Xu H Wang C Wei H Li T Fang Y Wang B . A novel missense variant in lamc1 identified in a poi family by whole exome sequencing. Gynecol Endocrinol. (2023) 39:2265507. doi: 10.1080/09513590.2023.2265507, PMID: 37839437

[79] Pyun JA Cha DH Kwack K . Lamc1 gene is associated with premature ovarian failure. Maturitas. (2012) 71:402–6. doi: 10.1016/j.maturitas.2012.01.011, PMID: 22321639

[80] Brown HM Dunning KR Robker RL Pritchard M Russell DL . Requirement for adamts-1 in extracellular matrix remodeling during ovarian folliculogenesis and lymphangiogenesis. Dev Biol. (2006) 300:699–709. doi: 10.1016/j.ydbio.2006.10.012, PMID: 17097630

[81] Richards JS . Ovulation: new factors that prepare the oocyte for fertilization. Mol Cell Endocrinol. (2005) 234:75–9. doi: 10.1016/j.mce.2005.01.004, PMID: 15836955

[82] Cookingham LM Van Voorhis BJ Ascoli M . Do alterations in follicular fluid proteases contribute to human infertility? J Assist Reprod Genet. (2015) 32:737–45. doi: 10.1007/s10815-015-0447-9, PMID: 25682117 PMC4429453

[83] Russell DL Doyle KM Ochsner SA Sandy JD Richards JS . Processing and localization of adamts-1 and proteolytic cleavage of versican during cumulus matrix expansion and ovulation. J Biol Chem. (2003) 278:42330–9. doi: 10.1074/jbc.M300519200, PMID: 12907688

[84] Shozu M Minami N Yokoyama H Inoue M Kurihara H Matsushima K . Adamts-1 is involved in normal follicular development, ovulatory process and organization of the medullary vascular network in the ovary. J Mol Endocrinol. (2005) 35:343–55. doi: 10.1677/jme.1.01735, PMID: 16216914

[85] Shindo T Kurihara H Kuno K Yokoyama H Wada T Kurihara Y . Adamts-1: A metalloproteinase-disintegrin essential for normal growth, fertility, and organ morphology and function. J Clin Invest. (2000) 105:1345–52. doi: 10.1172/jci8635, PMID: 10811842 PMC315464

[86] Menke DB Koubova J Page DC . Sexual differentiation of germ cells in xx mouse gonads occurs in an anterior-to-posterior wave. Dev Biol. (2003) 262:303–12. doi: 10.1016/s0012-1606(03)00391-9, PMID: 14550793

[87] Knauff EA Franke L van Es MA van den Berg LH van der Schouw YT Laven JS . Genome-wide association study in premature ovarian failure patients suggests adamts19 as a possible candidate gene. Hum Reprod. (2009) 24:2372–8. doi: 10.1093/humrep/dep197, PMID: 19508998

[88] Minns AF Qi Y Yamamoto K Lee K Ahnström J Santamaria S . The C-terminal domains of adamts1 contain exosites involved in its proteoglycanase activity. J Biol Chem. (2023) 299:103048. doi: 10.1016/j.jbc.2023.103048, PMID: 36813235 PMC10033314

[89] Chen K Yang K Luo SS Chen C Wang Y Wang YX . A homozygous missense variant in hsd17b4 identified in a consanguineous chinese han family with type ii perrault syndrome. BMC Med Genet. (2017) 18:91. doi: 10.1186/s12881-017-0453-0, PMID: 28830375 PMC5568266

[90] Tsui V Crismani W . The fanconi anemia pathway and fertility. Trends Genet. (2019) 35:199–214. doi: 10.1016/j.tig.2018.12.007, PMID: 30683429

[91] Kunz C Schär P . Meiotic recombination: sealing the partnership at the junction. Curr Biol. (2004) 14:R962–4. doi: 10.1016/j.cub.2004.10.043, PMID: 15556855

[92] Wyrwoll MJ van Walree ES Hamer G Rotte N Motazacker MM Meijers-Heijboer H . Bi-allelic variants in DNA mismatch repair proteins muts homolog msh4 and msh5 cause infertility in both sexes. Hum Reprod. (2021) 37:178–89. doi: 10.1093/humrep/deab230, PMID: 34755185

[93] Smirin-Yosef P Zuckerman-Levin N Tzur S Granot Y Cohen L Sachsenweger J . A biallelic mutation in the homologous recombination repair gene spidr is associated with human gonadal dysgenesis. J Clin Endocrinol Metab. (2017) 102:681–8. doi: 10.1210/jc.2016-2714, PMID: 27967308

[94] Palmer JS Zhao ZZ Hoekstra C Hayward NK Webb PM Whiteman DC . Novel variants in growth differentiation factor 9 in mothers of dizygotic twins. J Clin Endocrinol Metab. (2006) 91:4713–6. doi: 10.1210/jc.2006-0970, PMID: 16954162

[95] Bretherick KL Fairbrother N Avila L Harbord SH Robinson WP . Fertility and aging: do reproductive-aged canadian women know what they need to know? Fertil Steril. (2010) 93:2162–8. doi: 10.1016/j.fertnstert.2009.01.064, PMID: 19296943

[96] Chatterjee S Singh R Kadam S Maitra A Thangaraj K Meherji P . Longer cag repeat length in the androgen receptor gene is associated with premature ovarian failure. Hum Reprod. (2009) 24:3230–5. doi: 10.1093/humrep/dep296, PMID: 19684044

[97] Sugawa F Wada Y Maruyama T Uchida H Ishizuka B Ogata T . Premature ovarian failure and androgen receptor gene cag repeat lengths weighted by X chromosome inactivation patterns. Fertil Steril. (2009) 91:649–52. doi: 10.1016/j.fertnstert.2007.11.085, PMID: 18281036

[98] Chen L Bao BY Chang WC Ho JY Cheng BH Wang CL . Short androgen receptor poly-glutamine-promoted endometrial cancer is associated with benzo[a]Pyrene-mediated aryl hydrocarbon receptor activation. J Cell Mol Med. (2018) 22:46–56. doi: 10.1111/jcmm.13291, PMID: 28782227 PMC5742722

[99] Huang C Zhao S Yang Y Guo T Ke H Mi X . Tp63 gain-of-function mutations cause premature ovarian insufficiency by inducing oocyte apoptosis. J Clin Invest. (2023) 133. doi: 10.1172/jci162315, PMID: 36856110 PMC9974095

[100] Han J Hu Y Ding S Liu S Wang H . The analysis of the pyroptosis-related genes and hub gene tp63 cerna axis in osteosarcoma. Front Immunol. (2022) 13:974916. doi: 10.3389/fimmu.2022.974916, PMID: 36389801 PMC9664215

[101] Zhong Y Peng F Luo X Wang X Yang B Tang X . A pyroptosis-related gene signature for prognostic and immunological evaluation in breast cancer. Front Oncol. (2022) 12:964508. doi: 10.3389/fonc.2022.964508, PMID: 36936274 PMC10020702

[102] Bencivenga D Stampone E Azhar J Parente D Ali W Del Vecchio V . P27(Kip1) and tumors: characterization of cdkn1b variants identified in men4 and breast cancer. Cells. (2025) 14. doi: 10.3390/cells14030188, PMID: 39936980 PMC11817124

[103] Halperin R Arnon L Nasirov S Friedensohn L Gershinsky M Telerman A . Germline cdkn1b variant type and site are associated with phenotype in men4. Endocr Relat Cancer. (2023) 30. doi: 10.1530/erc-22-0174, PMID: 36256846

[104] Pan Y Zhang Q Tian L Wang X Fan X Zhang H . Jab1/csn5 negatively regulates P27 and plays a role in the pathogenesis of nasopharyngeal carcinoma. Cancer Res. (2012) 72:1890–900. doi: 10.1158/0008-5472.Can-11-3472, PMID: 22350412 PMC3460549

[105] Hou D Yao C Xu B Luo W Ke H Li Z . Variations of C14orf39 and syce1 identified in idiopathic premature ovarian insufficiency and nonobstructive azoospermia. J Clin Endocrinol Metab. (2022) 107:724–34. doi: 10.1210/clinem/dgab777, PMID: 34718620

[106] Ishida Y Kakiuchi N Yoshida K Inoue Y Irie H Kataoka TR . Unbiased detection of driver mutations in extramammary paget disease. Clin Cancer Res. (2021) 27:1756–65. doi: 10.1158/1078-0432.Ccr-20-3205, PMID: 33323405

[107] Ma L Chen Y Mei S Liu C Ma X Li Y . Single nucleotide polymorphisms in premature ovarian failure−Associated genes in a chinese hui population. Mol Med Rep. (2015) 12:2529–38. doi: 10.3892/mmr.2015.3762, PMID: 25954833 PMC4464472

[108] M'Baye M Hua G Khan HA Yang L . Rnai-mediated knockdown of inhbb increases apoptosis and inhibits steroidogenesis in mouse granulosa cells. J Reprod Dev. (2015) 61:391–7. doi: 10.1262/jrd.2014-158, PMID: 26063610 PMC4623144

[109] Richani D Constance K Lien S Agapiou D Stocker WA Hedger MP . Cumulin and fsh cooperate to regulate inhibin B and activin B production by human granulosa-lutein cells *in vitro*. Endocrinology. (2019) 160:853–62. doi: 10.1210/en.2018-01026, PMID: 30753406

[110] Ju A Cho Y-C Kim BR Park SG Kim J-H Kim K . Scaffold role of dusp22 in ask1-mkk7-jnk signaling pathway. PloS One. (2016) 11:e0164259. doi: 10.1371/journal.pone.0164259, PMID: 27711255 PMC5053508

[111] Pleasant V Boggan J Richards B Milliron KJ Purrington KS Simon M . Reclassification of variants of uncertain significance by race, ethnicity, and ancestry for patients at risk for breast cancer. Front Oncol. (2025) 15:1455509. doi: 10.3389/fonc.2025.1455509, PMID: 40040729 PMC11876048

[112] Thummala A Sudhakaran R Gurram A Mersch J Badalamenti A Gottaway G . Variant reclassification and recontact research: A scoping review. Genet Med Open. (2024) 2:101867. doi: 10.1016/j.gimo.2024.101867, PMID: 39669626 PMC11613892

[113] Walsh N Cooper A Dockery A O'Byrne JJ . Variant reclassification and clinical implications. J Med Genet. (2024) 61:207–11. doi: 10.1136/jmg-2023-109488, PMID: 38296635

