# Supplementary figures and images for: Identification of novel variants underlying non-syndromic primary ovarian insufficiency using a targeted NGS gene panel
Source: Front Endocrinol (Lausanne). 2025 Dec 11;16:1659701. doi: 10.3389/fendo.2025.1659701 (PMC12738175; doi:10.3389/fendo.2025.1659701)

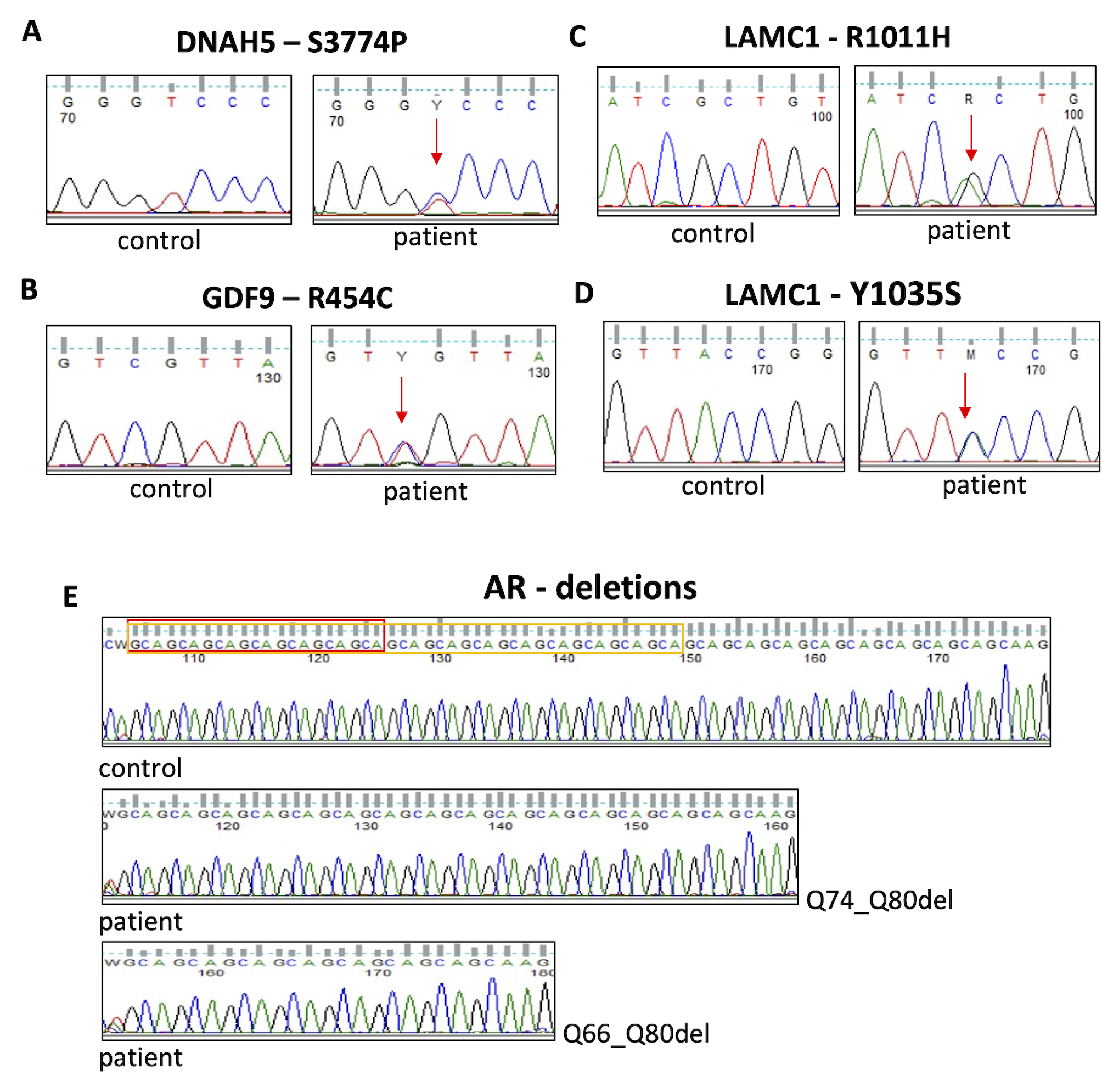

Supplement: Supplementary Figure 1 — Sanger sequencing analysis. Sanger sequencing was used to confirm the indicated mutations of DNAH5(A), GDF9(B), LAMC1(C, D), and AR(E) genes. [file Image1.tiff]

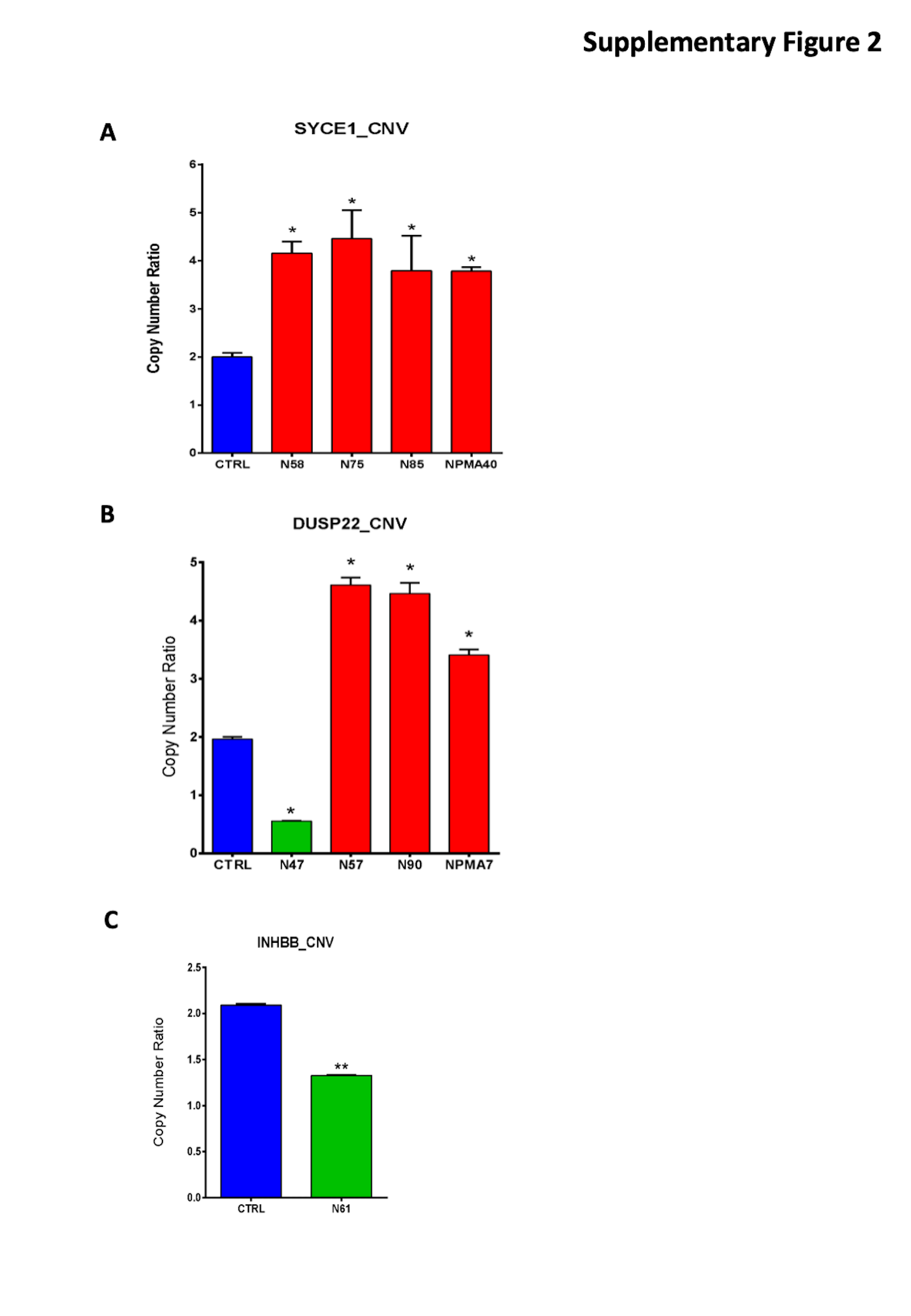

Supplement: Supplementary Figure 2 — Q-RT-PCR analysis. The graphs show the CN gains of SYCE1(A) and DUSP22(B) and the CN losses of INHBB(C) in each of the indicated patients. Values are expressed as CN ratios compared to a pool of control samples arbitrarily set as a reference for the diploid state (CN = 2). Statistical significance was calculated in relation to the pool of control samples: *p<0.05, **p<0.01. [file Image2.tiff]
